# Prolonged Adaptation to a Low or High Protein Diet Does Not Modulate Basal Muscle Protein Synthesis Rates – A Substudy

**DOI:** 10.1371/journal.pone.0137183

**Published:** 2015-09-14

**Authors:** Rick Hursel, Eveline A. P. Martens, Hanne K. J. Gonnissen, Henrike M. Hamer, Joan M. G. Senden, Luc J. C. van Loon, Margriet S. Westerterp-Plantenga

**Affiliations:** 1 Department of Human Biology, School of Nutrition and Translational Research in Metabolism, Maastricht University, Maastricht, The Netherlands; 2 Department of Human Movement Sciences, School of Nutrition and Translational Research in Metabolism Maastricht University, Maastricht, The Netherlands; The Chinese University of Hong Kong, HONG KONG

## Abstract

**Background:**

Based on controlled 36 h experiments a higher dietary protein intake causes a positive protein balance and a negative fat balance. A positive net protein balance may support fat free mass accrual. However, few data are available on the impact of more prolonged changes in habitual protein intake on whole-body protein metabolism and basal muscle protein synthesis rates.

**Objective:**

To assess changes in whole-body protein turnover and basal muscle protein synthesis rates following 12 weeks of adaptation to a low versus high dietary protein intake.

**Methods:**

A randomized parallel study was performed in 40 subjects who followed either a high protein (2.4 g protein/kg/d) or low protein (0.4 g protein/kg/d) energy-balanced diet (30/35/35% or 5/60/35% energy from protein/carbohydrate/fat) for a period of 12 weeks. A subgroup of 7 men and 8 women (body mass index: 22.8±2.3 kg/m^2^, age: 24.3±4.9 y) were selected to evaluate the impact of prolonged adaptation to either a high or low protein intake on whole body protein metabolism and basal muscle protein synthesis rates. After the diet, subjects received continuous infusions with L-[ring-^2^H_5_]phenylalanine and L-[ring-^2^H_2_]tyrosine in an overnight fasted state, with blood samples and muscle biopsies being collected to assess post-absorptive whole-body protein turnover and muscle protein synthesis rates *in vivo* in humans.

**Results:**

After 12 weeks of intervention, whole-body protein balance in the fasted state was more negative in the high protein treatment when compared with the low protein treatment (-4.1±0.5 vs -2.7±0.6 μmol phenylalanine/kg/h;P<0.001). Whole-body protein breakdown (43.0±4.4 vs 37.8±3.8 μmol phenylalanine/kg/h;P<0.03), synthesis (38.9±4.2 vs 35.1±3.6 μmol phenylalanine/kg/h;P<0.01) and phenylalanine hydroxylation rates (4.1±0.6 vs 2.7±0.6 μmol phenylalanine/kg/h;P<0.001) were significantly higher in the high vs low protein group. Basal muscle protein synthesis rates were maintained on a low vs high protein diet (0.042±0.01 vs 0.045±0.01%/h;P = 0.620).

**Conclusions:**

In the overnight fasted state, adaptation to a low-protein intake (0.4 g/kg/d) does not result in a more negative whole-body protein balance and does not lower basal muscle protein synthesis rates when compared to a high-protein intake.

**Trial Registration:**

Clinicaltrials.gov NCT01551238.

## Introduction

High-protein diets have attracted interest for many years because of their ability to preserve fat free mass (FFM) during negative energy balance [1, 2]. While being in a neutral or positive energy balance, a temporary increase in dietary protein consumption for 3 months can lead to an increase in FFM [3, 4], especially when combined with regular exercise [5]. Therefore, a temporary increase in dietary protein intake may act as a preventive measure to remain weight stable [6]. However, the impact of prolonged adaptation to a low or high protein intake on whole-body protein balance or muscle protein synthesis (MPS) has not been assessed. An increase in protein synthesis, accompanied by a simultaneous reduction in protein breakdown, due to increased protein consumption may be responsible for the preservation or increase of FFM, irrespective of energy balance.

Several studies [7–12] have shown that ingestion of dietary protein stimulates net muscle protein accretion. The post-prandial rise in circulating essential amino acids (EAA), and leucine in particular, has been identified as the key factor stimulating the post-prandial rise in MPS rate [8, 9]. In contrast to consumption of a high dietary protein diet, it is thought that a relatively low protein intake may lead to a decline in muscle protein synthesis, resulting in net protein loss. A diet providing 15 energy% protein, or an absolute amount of 0.8 g protein/kg/d, is recommended to maintain proper protein balance [13, 14]. Prolonged under-consumption of dietary protein has been suggested to induce muscle mass and strength loss. Nevertheless, despite a large amount of short-term studies investigating the impact of dietary protein consumption on whole-body protein turnover and MPS, few studies have examined the impact of prolonged adaptation to either a low or high protein intake on whole-body protein turnover and basal muscle protein synthesis rates. In the present substudy, we tested our hypothesis that consuming a diet low in dietary protein induces a negative whole-body protein balance and reduces basal muscle protein synthesis rates when compared with a high protein diet. We applied contemporary stable isotope methodology to assess the impact of a low versus high protein intake diet post-absorptive whole-body protein balance and fasting MPS rates *in vivo* in humans. Furthermore, we assessed 24 h whole-body protein balance by nitrogen balance following prolonged adaptation to a low and high protein intake diet.

## Materials and Methods

### Subjects

**Fig 1:**
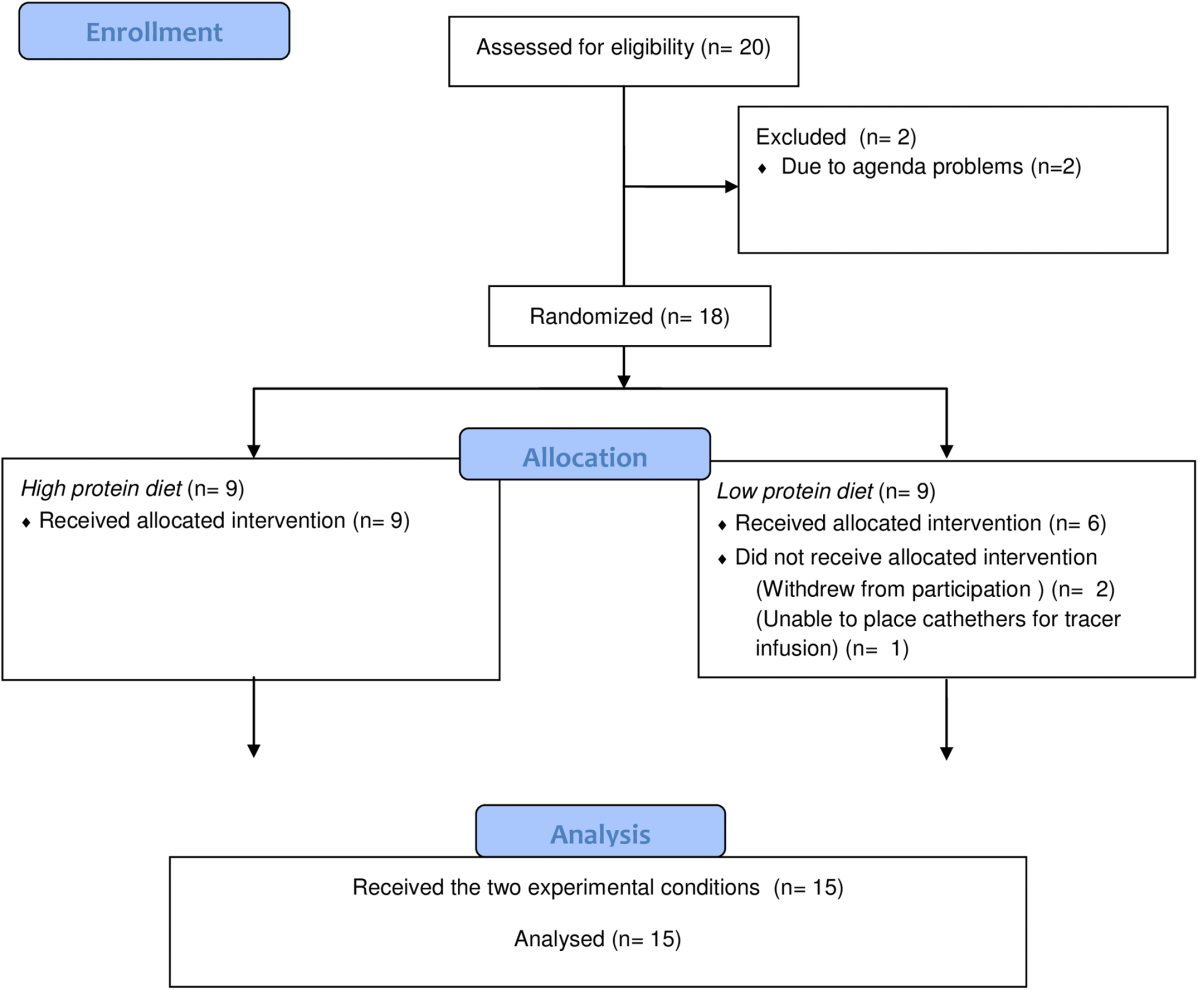
Flow diagram (CONSORT) of a substudy in two diet groups (12 weeks High Protein or Low Protein diet), with n = 20 eligible men and women.

In the main study, Martens *et al*. (*n* = 37) [15] studied the impact of a high-protein (HP) diet compared with a low-protein (LP) diet on satiety and energy expenditure. They concluded that maintenance of energy expenditure, initial transient higher fullness, and a sustained positive protein balance on a HP vs LP diet in energy balance may prevent development of a positive energy balance and subsequent body weight gain. In a subgroup of subjects (*n* = 20) we determined post-absorptive whole-body protein metabolism and basal muscle protein synthesis rates to assess possible changes following 12 weeks of adaptation to a high vs low dietary protein intake. After drop-out of 5 subjects (2 subjects due to agenda problems, one subject we were not able to place the catheters and 2 subjects withdrew from the study without giving any reason), 15 subjects, (7 men and 8 women), aged 19 to 31 y; body mass index between 19 and 26 kg/m^2^) participated in the current substudy **(Fig 1)**. The sample-size calculation was based on previous research from this lab on protein metabolism [16]. We calculated the sample size by using the following variables: the difference in fractional synthetic rate (FSR) of mixed muscle protein >20% and a SD of 15% with a type I error of 5% and a type II error of 10%. Power calculations showed that ≥ 9 subjects were needed, and therefore we decided to include 10 subjects per group. In the reference study, a crossover design with 10 healthy subjects was performed and resulted in significant effects of dietary protein consumption on muscle protein synthesis.

Subjects were recruited via advertisements in local newspapers and on notice boards at the university. Subject recruitment started in November 2012 and the study was conducted between January 2013 and September 2013. Subjects underwent a screening and all were in good health, non-smokers, not using medication (except for oral contraception) and moderate alcohol users (<10 drinks per week). None of them had a food allergy, gained or lost more than 3 kg in 6 months prior to the study, or were cognitive dietary restrained (F1>9) as assessed by a validated Dutch translation of the Three Factor Eating Questionnaire [17]. The validated Dutch translation of the Baecke Activity Questionnaire was used to measure habitual physical activity [18]. All procedures involving human subjects in this study, that were performed with a subgroup of subjects from the main study [15], were specifically approved by The Medical Ethical Committee of Maastricht University Medical Centre. This study was also conducted according to guidelines laid down in the Declaration of Helsinki. All subjects provided written informed consent. The main study [15] was registered at clinicaltrials.gov with identifier NCT01551238. The protocol for this trial and supporting CONSORT checklist are available as supporting information; see S1 Protocol and S1 CONSORT Checklist.

### Study design

The study had a randomized, single-blinded, parallel design and consisted of a long-term (12 weeks) dietary intervention. Subjects were randomly divided in two groups that received either a HP (2.4 g protein/kg/d, 30/35/35% of energy from protein/carbohydrate/fat) or a LP energy-balanced diet (0.4 g protein/kg/d, 5/60/35% of energy from protein/carbohydrate/fat).

In order to maintain their diet at home, all subjects received a booklet containing individual guidelines with permitted and non-permitted foods and their corresponding portions, as well as three example menus. The example menus consisted of commercially available food products and were tailored to the energy requirements of each subject based on basal metabolic rate calculated with the Harris and Benedict equation [19] and multiplied with a physical activity level of 1.7 estimated by means of a computer simulation program [20]. At week 5 and 9 of the intervention period subjects visited the university to discuss the compliance to the dietary guidelines with a researcher. Subjects on the HP diet (30% of energy from protein) had to drink two protein shakes each day during 12 weeks. The supplemental protein was whey protein with α-lactalbumin (Hiprotal Whey Protein Alpha and Domo; FrieslandCampina). Subjects on the LP diet (60% of energy from carbohydrate) had to drink two carbohydrate shakes each day during 12 weeks. The supplemental carbohydrate was maltodextrin (Fantomalt, Nestle). During screening subjects had to rate the palatability of the shakes. Only subjects who rated the shakes as sufficiently palatable (VAS score ≥ 50 mm), and who were confident of being able to consume these daily during the study period were included in the study. The fat content between conditions was maintained at a constant proportion (35% of energy from fat). Subjects were instructed to keep their body weight stable. Subjects’ characteristics reported in **Table 1**were determined as described by Martens *et al*. [15].

**Table 1:** Subjects’ characteristics.

	Δ	HP	LP	Total	P-Value
**N (M/F)**		9 (4/5)	6 (3/3)	15 (7/8)	
**Age (Y)**		23.9±4.2	25.0±6.2	24.3±4.9	0.686
**Height (m)**		1.70±0.08	1.70±0.09	1.70±0.09	0.899
**Weight (kg)**		62.8±6.1	67.3±8.6	65.1±7.1	0.312
	**Δ Weight (kg)**	+0.71±0.8	+0.06±1.2	+0.45±0.98	0.216
**BMI (kg/m** ^**2**^ **)**		22.1±2.4	23.3±2.2	22.6±2.3	0.373
	**Δ BMI (kg/m** ^**2**^ **)**	+0.26±0.30	+0.04±0.39	+0.17±0.34	0.903
**FM%**		24.2±7.3	22.7±7.8	23.6±7.3	0.339
	**Δ FM (%)**	+0.04±1.31	+0.32±0.97	+0.15±1.16	0.672
**FFM%**		75.8±7.3	77.4±7.8	76.5±7.3	0.709
	**Δ FFM (%)**	-0.04±1.31	-0.32±0.97	-0.15±1.16	0.672
**PAL**		1.82±0.14	1.79±0.15	1.81±0.14	0.677

Δ changes over 12 weeks. BMI, Body Mass Index; FM, fat mass; FFM, fat free mass; PAL, physical activity level. These data concern the as analysed population and not the as randomised population. Values are expressed as mean ± SD. Data were analyzed with one-way ANOVA. Table adapted and modified from Martens et al. [15].

### Biomarker of protein intake and 24-h protein turnover

Nitrogen excretion was used as biomarker for protein intake (to measure compliance) and to estimate 24-h protein turnover. Subjects collected their 24-h urine at five different time points during the 12 week period. Collection started after the first voiding in the morning at 0800h and lasted until the next day at 0800h including the first voiding. The total volume of the 24-h urine was recorded. Urine was collected in 2-L urine bottles with 10 mL of diluted hydrochloric acid (4 mmol/L) added to prevent nitrogen loss through evaporation. Urine was gently mixed, and samples were taken and stored at -20°C until analysis. Nitrogen concentrations were measured with a nitrogen analyzer (CHO-O-Rapid; Hereaus). 24-h protein turnover was calculated using the prescribed protein intake and measured urinary nitrogen excretion data.

### Test day

The experiment started at 0800 am, when overnight-fasted subjects arrived at the laboratory by car or public transportation. A polytetrafluoroethylene catheter was inserted into an antecubital vein for stable isotope infusion. A second polytetrafluoroethylene catheter was inserted in a heated dorsal hand vein of the contralateral arm and placed in a hot box (60°C) for arterialized blood sampling [21]. After basal blood collection (t = 0 min), plasma phenylalanine (Phe) and tyrosine (Tyr) pools were primed with a single intravenous dose of L-[ring-^2^H_5_] Phe (2 *μ*mol/kg) and L-[ring-^2^H_2_]Tyr (0.615 *μ*mol/kg), after which a continuous L-[ring-^2^H_5_]Phe and L-[ring-^2^H_2_]Tyr infusion was started (0.050±0.0005 and 0.015±0.0001 *μ*mol/kg/min, respectively). After resting in a supine position for 120 min, a second arterialized blood sample was drawn, and a muscle biopsy was collected from the *vastus lateralis* muscle (t = 120 min). Additional arterialized blood samples (8 mL) were collected at t = 180, 240, and 300 min with a second muscle biopsy, taken from the contralateral leg, at t = 300 min, which marked the end of the basal fasting period as well as the experiment. Blood samples were collected in tubes containing EDTA and centrifuged at 1000*g* for 10 min at 4°C. Aliquots of plasma were frozen in liquid nitrogen and stored at –80°C. Muscle biopsies were obtained from the middle region of the *vastus lateralis*, 15 cm above the patella and ~3 cm below entry through the fascia, by using the percutaneous needle biopsy technique [22]. Muscle samples were dissected carefully and freed from any visible non-muscle material. Muscle samples were immediately frozen in liquid nitrogen and stored at –80°C until additional analysis.

### Plasma analysis

Plasma glucose (Uni Kit III, 07367204; Roche) concentrations were analyzed with a COBAS-FARA semiautomatic analyzer (Roche). Insulin was analyzed by using a radioimmunoassay (Insulin RIA kit; LINCO Research Inc). Plasma (100 mL) for amino acid analyses was deproteinized on ice with 10 mg dry 5-sulphosalicylic acid, mixed, and the clear supernatant fluid was collected after centrifugation. Plasma amino acid concentrations were determined by using HPLC after precolumn derivatization with *o*-phthaldialdehyde [23]. For plasma enrichment measurements, plasma Phe and Tyr were derivatized to their *t*-butyldimethylsilyl derivatives and analyzed by using gas chromatography–mass spectrometry (GC-MS) (Agilent 6890N GC/5973N MSD; Agilent) by using selected ion monitoring of masses 336 and 341 for unlabeled and labeled (ring-^2^H_5_) Phe, respectively; and masses 466, 468, and 470 for unlabeled and labeled (ring-^2^H_2_ and ring-^2^H_4_) Tyr, respectively [24]. Thereafter, ratios of labeled: unlabeled derivatives were analyzed by using gas chromatography–combustion isotope ratio mass spectrometry (FinniganMAT 252; ThermoFisher Scientific). Standard regression curves were applied in all isotopic enrichment analyses to assess the linearity of the mass spectrometer and to control for the loss of tracer.

### Muscle analysis

For the measurement of L-[ring-^2^H_5_]Phe enrichment in the muscle tissue–free amino acid pool and mixed muscle protein, 55 mg wet muscle was freeze-dried. Collagen, blood, and other non-muscle fiber material were removed from muscle fibers under a light microscope. The isolated muscle fiber mass (10–15 mg) was weighed, and 8 volumes (8x dry weight of isolated muscle fibers x wet:dry ratio) of ice-cold 2% perchloric acid were added. The tissue was homogenized and centrifuged. The supernatant fluid was collected and processed in the same manner as plasma samples, such that tissue-free L-[ring-^2^H_5_]Phe enrichments could be measured by using their *t*-butyldimethylsilyl derivatives on a GC-MS.

The protein pellet was washed with 3 additional 1.5-mL washes of 2% perchloric acid, dried, and hydrolyzed in 6 mol/L HCl at 120°C for 15–18h. The hydrolyzed protein fraction was dried under a nitrogen stream while heated to 120°C, and a 50% acetic acid solution was added, and the hydrolyzed protein was passed over a Dowex exchange resin (AG 50W-X8, 100–200 mesh hydrogen form; Biorad) by using 2 mol/L NH_4_OH. The eluate was collected, and L-[ring-^2^H_5_]Phe was derivatized to N-methyl-N-tert-butyldimethylsilyltrifluoroacetamidephenylethyl- amine [25]. Thereafter, ratios of labeled:unlabeled derivatives were determined by using GC-MS. Standard regression curves were applied to assess the linearity of the mass spectrometer and to control for the loss of tracer.

### Calculations

The intravenous infusion of L-[ring-^2^H_5_]Phe and L-[ring-^2^H_2_]Tyr, and arterialized blood sampling were used to assess whole-body protein metabolism in steady state conditions. The total Phe rate of appearance (Ra) was calculated by using modified Steele’s equations [26, 27]. These variables were calculated as follows:

$$(1)\;\text{Total}R_{\text{a}}=(F-(\text{pV}\;\text{x}\;C\left(\text{t}\right)\;\text{x}\;{\text{dE}}_{\text{iv}}/\text{dt}))/{\text{E}}_{\text{iv}}\left(\text{t}\right)=\text{protein breakdown}$$

where *F* is the intravenous tracer infusion rate (*μ*mol/kg/min), pV (0.125) is the distribution volume for Phe [27]. *C*(*t*) is the mean plasma Phe concentration between two consecutive time points. d*E*_iv_/dt represents the time-dependent variations of plasma Phe enrichment derived from the intravenous tracer, and *E*_iv_(*t*) is the mean plasma Phe enrichment from the intravenous tracer between 2 consecutive time points. Total *R*_a_ represents the plasma entry of Phe derived from whole-body protein breakdown. The total rate of disappearance of Phe (total *R*_d_) equals the Phe-to-Tyr conversion rate (first step in Phe oxidation) and utilization for protein synthesis. These variables were calculated as follows:

$$\left(2)\;\text{Total}\;R_{\text{d}}=\text{total}\;R_{\text{a}}-(\text{pV}\;\text{x}\;\text{dC}/\text{dt}\right)$$

$$\left(3)\;\text{PHE to TYR conversion}=\text{Tyr}R_{\text{a}}\;\text{x}\;((E_{\text{t}}(\text{t}))/(E_{\text{p}}(\text{t})))\;\text{x}\;(\text{Phe}R_{\text{d}}/(F\text{p}+\text{Phe}R_{\text{d}})\right)$$

$$(4)\;\text{Protein synthesis}=\text{total}\;R_{\text{d}}-\text{PHE to TYR conversion}$$

$$(5)\;\text{PHE net balance}=\text{protein synthesis}-\text{Total}\;R_{\text{a}}$$

where Phe *R*_d_ and Tyr *R*_a_ are the flux rates for Phe and Tyr, respectively; *E*_t_(*t*) and *E*_p_(*t*) are the mean plasma enrichments of L-[ring-^2^H_2_]Tyr and L-[ring-^2^H_5_]Phe, respectively; and *F*_p_ is the infusion rate of the Phe tracer. The FSR (in %/h) was calculated by using the precursor-product method [24]:

$$(6)\;\text{FSR}=(\Delta E_{\text{p}}/(E_{\text{precursor}}\;\text{x}\;t))\;\text{x}\;100$$

where Δ*E*_p_ is the Δ increment of muscle protein-bound L-[ring-^2^H_5_]Phe during the incorporation period. *E*_precursor_ is the average plasma L-[ring-^2^H_5_]Phe enrichment during the time period for determination of amino acid incorporation, and *t* indicates the time interval (h) between biopsies.

### Statistical analysis

A one-way ANOVA was used to assess differences in subject characteristics, hormone, amino acid concentrations and basal FSRs between treatments. Further, a 2-factor ANOVA with time as factor 1 and treatment as factor 2 was used to assess differences between treatments over time (time x treatment interaction) for plasma Phe, Tyr, Leu concentrations and plasma enrichments, as well as whole-body protein metabolism. In case of a significant time x treatment interaction, pairwise comparisons for individual time points were applied to locate differences between treatments. A 2-factor repeated measures ANOVA was used to assess differences over time and between treatments for 24-h protein turnover (protein intake, excretion and balance). Finally, Gender comparisons for whole-body protein metabolism and basal FSRs were made using a 2-factor ANOVA with gender as factor 1 and treatment as factor 2 (gender x treatment interaction). Statistical significance was set at P<0.05. All calculations were performed with the SPSS 20.0 software package (SPSS Inc). All data are expressed as means ± SEMs. For whole-body protein metabolism, FSR and 24 h protein turnover data we also report the 95% confidence interval as well as the mean difference, SEMs and the 95% confidence interval of the mean difference.

## Results

All individual data points behind means, medians and variance measures presented in the results, tables and figures are available in **S1 File**.

### Plasma analyses

Basal plasma insulin (10.9 ± 1.8 vs 11.0 ± 1.7 mU/l; P = 0.879) and glucose (4.95 ± 0.18 vs 4.62 ± 0.11 mmol/l; P = 0.153) concentrations did not differ between the HP vs LP group after 12 weeks.

**Fig 2:**
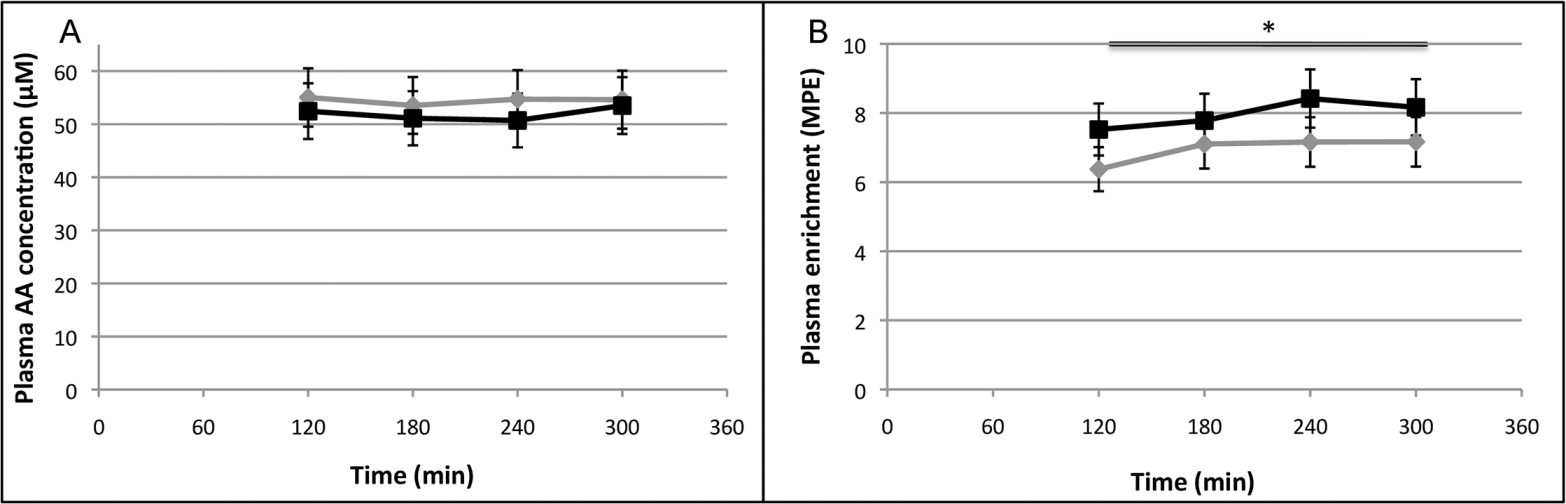
Mean (+SEM) plasma Phe (A) concentration (μmol L^-1^) and plasma L-[ring-^2^H_5_]Phe (B) enrichment (MPE) after 12 weeks on a HP (grey) vs LP (black) diet. *n* = 15. Data were analyzed with a two-factor ANOVA (time x treatment). *Treatment effect P<0.001.

Plasma Phe is illustrated over time in **Fig 2A**. After 12 weeks on a HP vs LP diet plasma Phe levels were not significantly different between groups (P = 0.282). Plasma Tyr (P<0.05) and Leu (P<0.03) concentrations were significantly increased after the HP diet compared with the LP diet. At week 12, basal EAA (P = 0.551), basal branched-chain amino acids (P = 0.402) and basal total amino acids (P = 0.535) concentrations in plasma were not different between the HP vs LP group (**Table 2**).

**Table 2:** Hormone and amino acids concentrations in plasma at baseline.

	HP	LP	P-value
**Glucose (mmol L** ^**-1**^ **)**	4.9 ± 0.2	4.6 ± 0.1	0.948
**Insulin (mU L** ^**-1**^ **)**	10.9 ± 1.8	11.0 ± 1.7	0.202
**Phenylalanine (μmol L** ^**-1**^ **)**	55 ± 2	52 ± 4	0.121
**Tyrosine (μmol L** ^**-1**^ **)**	54 ± 3	46 ± 3	<0.01
**Leucine (μmol L** ^**-1**^ **)**	122 ± 5	105 ± 8	<0.01
**Total BCAA (μmol L** ^**-1**^ **)**	457 ± 42	374 ± 41	0.199
**Total EAA (μmol L** ^**-1**^ **)**	969 ± 75	880 ± 54	0.402
**Total AA (μmol L** ^**-1**^ **)**	2566 ± 148	2642 ± 117	0.717

BCAA, branched chain amino acids; EAA, essential amino acids; AA, amino acids. Values are expressed as mean ± SEM. Data were analyzed with one-way ANOVA.

**Fig 2B** shows the time course of the plasma L-[*ring* ^2^H_5_]phenylalanine enrichment. Plasma L-[*ring* ^2^H_5_]phenylalanine enrichments were significantly higher in the LP group compared with the HP group (P<0.001).

### Whole-body protein metabolism

**Fig 3:**
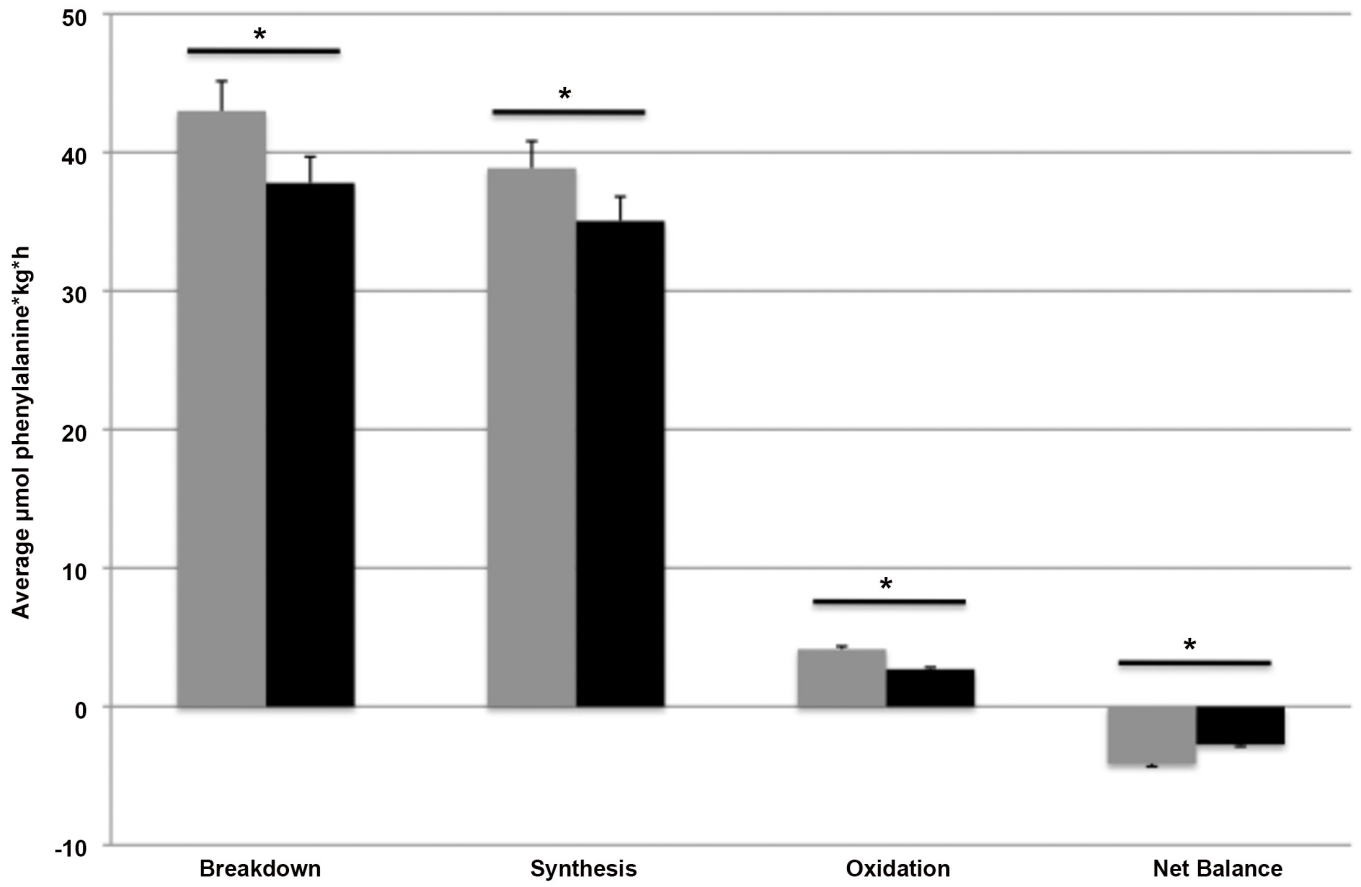
Mean (+SEM) basal whole-body protein metabolism expressed as the AUC (μmol Phe/kg/h) after 12 weeks on a HP (grey) vs LP (black) diet. n = 15. Data were analyzed with a two-factor ANOVA (time x treatment). *Treatment effect P<0.001.

Whole-body protein synthesis, breakdown, oxidation and net balance in the fasted state are expressed as the AUC in **Fig 3**. Whole-body protein synthesis, reflected by Phe-utilization and expressed as the average of total Phe R_d_ minus the conversion rate of Phe into Tyr, was significantly higher in the HP group (38.9±4.2 μmol Phe/kg/h, 95%CI: 36.1–42.0; P<0.01) vs LP group (35.1±3.6 μmol Phe/kg/h, 95%CI: 31.5–38.6; P<0.01), with a mean difference of 4.01±2.10 μmol Phe/kg/h, 95%CI: -0.60–8.63; P = 0.008. Phenylalanine R_d_ was also increased in the HP-group (43.0±4.4 μmol Phe/kg/h, 95%CI: 40.3–46.1; P<0.03) vs the LP group (37.8±3.8 μmol Phe/kg/h, 95%CI: 34.3–41.3; P<0.03), with a mean difference of 5.44±2.08 μmol Phe/kg/h, 95%CI: 0.86–10.02; P = 0.024.

Whole-body protein oxidation, which was expressed as the average of the Phe-to-Tyr conversion rate, was greater in the HP group (4.1±0.6 μmol Phe/kg/h, 95%CI: 3.85–4.53; P<0.001) vs the LP group (2.7±0.6 μmol Phe/kg/h, 95%CI: 2.29–3.11; P<0.001), with a mean difference of 1.49±0.24 μmol Phe/kg/h, 95%CI: 0.96–2.03; P = 0.000. The deduction of synthesis minus breakdown, resulting in whole-body protein balance, was more negative in the HP group (-4.1±0.5 μmol Phe/kg/h, 95%CI: -4.50 –-3.84; P<0.001) compared with the LP group (-2.7±0.6 μmol Phe/kg/h, 95%CI: -3.14 –-2.34; P<0.001), with a mean difference of -1.43±0.24 μmol Phe/kg/h, 95%CI: -1.95 –-0.91; P = 0.000.

Whole-body protein metabolism differed significantly between men and women with higher protein synthesis (P<0.05), protein breakdown (P<0.01) and protein oxidation (P<0.001) rates as well as a more negative protein net balance (P<0.001) in men compared with women. When expressed per kg FFM, only protein synthesis and protein breakdown rates differed between genders, with higher protein synthesis (P<0.03) and protein breakdown (P = 0.05) rates for women. There were no significant interactions between gender and treatment for whole-body protein metabolism

### Muscle tracer analysis and mixed-muscle protein synthesis rates

The increment in muscle protein bound L-[ring-^2^H_5_]Phe enrichment between the first and the second biopsy did not differ between the HP group and the LP group (0.0094±0.0023 vs 0.0101±0.032 MPE; P = 0.395).

**Fig 4:**
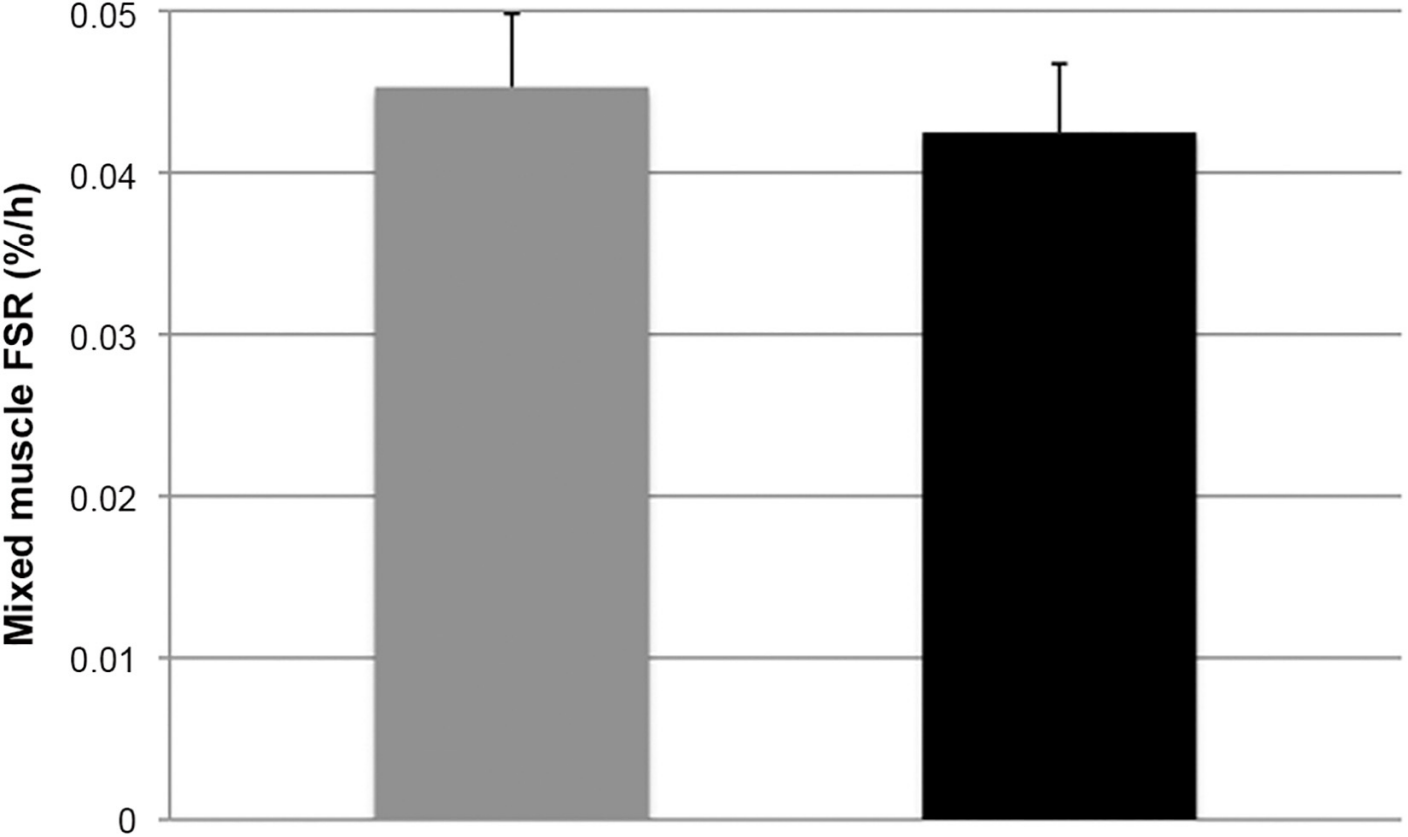
Mean (+SEM) mixed muscle protein fractional synthesis rates (%/h) in the basal state after 12 weeks on a HP (grey) vs LP (black) diet. n = 15. Data were analyzed with a one-way ANOVA.

Post-absorptive mixed MPS rates **(Fig 4)** did not differ following 12 weeks on either the HP diet (0.042±0.01%/h, 95%CI: 0.039–0.051; P = 0.620) or LP diet (0.045±0.01%/h, 95%CI: 0.035–0.050; P = 0.620), with a mean difference of 0.002±0.004%/h, 95%CI: -0.007–0.012; P = 0.621. FSRs were significantly higher in women vs men (P<0.01), without any significant interaction for gender vs treatment.

### 24h-nitrogen retention

**Fig 5:**
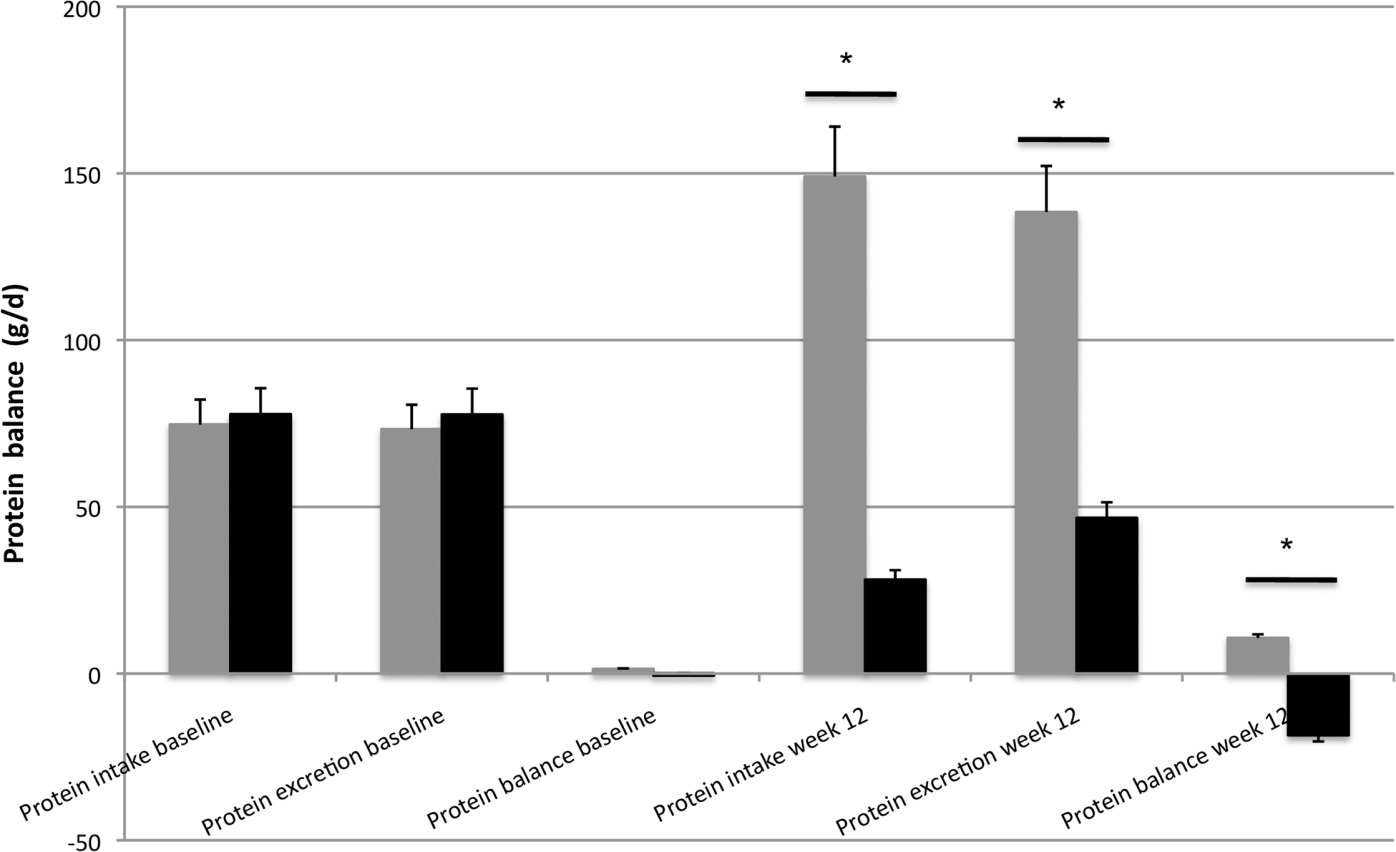
Mean (+SEM) protein intake, excretion and balance (g/d) at baseline and after 12 weeks on a HP (grey) vs LP (black) diet. n = 15. Data were analyzed with a two-factor repeated measures ANOVA (time x treatment). *Treatment effect P<0.001. #Time effect P<0.03. Adapted and modified from Martens et al. [15].

In **Fig 5**we present results for this subgroup, taken from Martens et al. [15] providing estimates of 24h- whole body protein turnover, based on nitrogen retention data. 24h-protein turnover, based on protein intake and nitrogen excretion, did not differ between the HP (0.1 ± 7.7 g/d, 95%CI: -11.7–12.7; P = 0.892) vs LP (1.4 ± 5.1 g/d, 95%CI: -14.7–15.0; P = 0.892) diet groups at baseline, with a mean difference of 0.41 ± 8.7 g/d, 95%CI: -18.8–19.6; P = 0.964. During the following 12 weeks, protein intake and nitrogen excretion were significantly increased in the HP (P<0.001) and significantly decreased in the LP (P<0.03) diet group compared with baseline. Therefore, 24h-protein turnover at week 12 was significantly different between the HP (10.7 ± 5.2 g/d, 95%CI: -0.50–21.2; P<0.01) vs LP diet groups (-18.5 ± 4.4 g/d, 95%CI: -31.7 –-5.3; P<0.01), with a mean difference of 28.9 ± 7.7 g/d, 95%CI: 11.8–46.0; P = 0.003. Moreover, the observed change between baseline and week 12 was significantly different between the HP (9.3 ± 5.9 g/d, 95%CI: -4.2–23.8; P<0.03) and the LP treatment (-18.6 ± 8.5 g/d, 95%CI: -35.7 –-1.6; P<0.03), with a mean difference of 28.5 ± 10.0 g/d, 95%CI: 6.4–50.5; P = 0.016.

Protein intake (P<0.001) and nitrogen excretion (P<0.01) were significantly different between men and women. However, 24h-protein turnover (P = 0.485) as well as the changes over time (P = 0.626) did not differ between both genders

## Discussion

In the current study, continuous intravenous infusions of L-[ring-^2^H_5_]Phenylalanine and L-[ring-^2^H_2_]Tyrosine were used to asses whole-body protein metabolism and MPS rates in the basal fasted state, in subjects that followed either a HP diet or LP diet for 12 weeks. Prolonged adaptation to a low dietary protein intake may lead to muscle loss due to a negative whole-body protein balance and a decline in basal muscle protein synthesis rates [13]. The results from the present study show that basal whole-body protein synthesis, breakdown, and oxidation rates were modified following three months on a LP when compared with a HP diet. Protein turnover was significantly lower with significant lower protein synthesis, protein breakdown, and protein oxidation rates on the low protein intake diet. Despite the lower protein turnover rates, post-absorptive whole-body net protein balance had not declined following prolonged adaptation to the low when compared with the high protein intake diet **(Fig 3)**.

Post-absorptive plasma phenylalanine kinetics can be used to provide some insight in whole-body protein turnover rates. When combined with the intravenous infusion of a tyrosine tracer, phenylalanine oxidation rates can be assessed via the rate of phenylalanine hydrolysis to tyrosine [28, 29]. Despite obvious limitations [28, 29], phenylalanine kinetics provide us with a good estimate of potential differences in basal protein breakdown, synthesis, and oxidation rates on a whole body level. However, these measurements of basal protein synthesis and protein breakdown rates do not necessarily reflect skeletal muscle tissue protein metabolism. Therefore, we also collected multiple muscle tissue biopsies to measure fasting mixed muscle protein fractional synthesis rates. In line with the absence of a decline in post-absorptive whole-body protein balance following adaptation to a low protein intake, we did not detect a decline in basal mixed muscle protein synthesis rates when comparing muscle protein synthesis rates after the low protein versu high protein intake diet. To our surprise, prolonged habituation to a diet providing only 0.4 g protein per kg body mass per day did not lower basal muscle protein synthesis rates, with muscle protein synthesis rates being maintained at the same level observed in the high protein intake diet **(Fig 4)**. The observation that post-absorptive whole-body protein balance as well as mixed muscle protein synthesis rates were not reduced on the low protein intake regimen seems to be in line with the observations in the full study, in which no changes in body composition were observed following 12 weeks of adaptation to a low or high protein intake with respect to body weight, FFM and fat mass. Apparently, prolonged adaptation to a low protein intake reduces whole-body protein turnover but does not seem to compromise whole-body protein balance, basal muscle protein synthesis rates or skeletal muscle mass maintenance. This study is the first to show that on a low protein diet (0.4 g/kg/d) body mass and fat free mass can be preserved by lowering whole-body turnover and maintaining basal muscle protein turnover rates. Future work is required to investigate the factors responsible for orchestrating such a protein conservation strategy.

Though we show that prolonged adaptation to a (very) low protein intake diet (0.4 g/kg/d) allows maintenance of post-absorptive whole body protein balance as well as basal muscle protein synthesis rates, we can only speculate on the impact of prolonged adaptation to a low compared with a high protein intake on 24 h whole-body protein balance as well as post-prandial muscle protein synthesis rates. It could be speculated that a low versus high protein intake diet modulates dietary protein digestion and amino acid absorption, peripheral amino acid uptake, muscle protein synthesis and breakdown in an effort to maintain a proper post-prandial muscle protein synthetic response to feeding and, as such, allow skeletal muscle maintenance. In the full study [15] we performed 24 h nitrogen balance analyses as a means to verify adherence and compliance to the low and high protein intake diet. We could use these data to get some insight in the overall 24 h whole-body protein balance following ingestion of a low or high protein diet. The 24 h nitrogen excretion data revealed a more positive whole-body protein balance in the HP versus LP diet [15], suggesting greater tissue protein accretion following the high protein intake diet. However, this seems to be at odds with the absence of any significant changes in body mass or fat free mass over the 12 week time span in both dietary conditions [15]. However, the more positive whole-body protein balance on the high protein versus low protein intake diet when assessed using 24 h nitrogen balance is likely attributed to the applied methodology. Previous work has already shown implausible high nitrogen retention rates when ingesting large amounts of protein [30–32].

Evidently, nitrogen retention data should be interpreted with much caution when using them as a proxy for changes in whole-body or muscle protein metabolism. Clearly, more work is required to assess whether the maintenance of post-absorptive whole body protein balance and basal muscle protein synthesis rates are accompanied by changes in the (muscle) protein synthetic response to feeding [33].

Though we did not intend to assess potential gender differences in basal protein balance and fasting MPS rates, we did observe differences with respect to whole-body protein balance and mixed muscle protein synthesis rates between men and women. Despite the small number of men and women, our data show that post-absorptive whole-body and muscle protein synthesis rates where greater in women compared with men after correction for differences in fat free mass. This seems to be in line with some [34] but certainly not all researchers, who generally fail to detect any major gender differences with respect to post-absorptive muscle protein synthesis rates [35–38].

In conclusion, prolonged adaptation to a low dietary protein intake lowers fasting, whole-body protein turnover rates, but does not compromise post-absorptive whole-body net protein balance. Post-absorptive skeletal muscle protein synthesis rates are maintained even when consuming a (very) low protein intake diet (0.4 g/kg/d).

## Supporting Information

S1 CONSORT Checklist CONSORT checklist. (DOCX)

S1 Protocol Study protocol containing background, hypothesis, outcome parameters and experimental design. (PDF)

S1 File Data file which contains all the individual data points behind means, medians and variance measures presented in the results, tables and figures. Data has been presented in HP and LP diet groups and we also discriminated between males and females. Subject codes have been removed to ensure privacy. Tab 1 contains the data of the plasma analyses. Tab 2 and 3 contain the whole-body kinetics. Tab 4 contains the eventual outcomes of the whole-body protein metabolism. Tab 5 contains the muscle biopsy data the muscle tracer analysis and mixed-muscle protein synthesis rates. Tab 6 contains the 24 h protein turnover data.
(XLSX)
