# Supplementary material for: Prolonged Adaptation to a Low or High Protein Diet Does Not Modulate Basal Muscle Protein Synthesis Rates – A Substudy
Source: PLoS One. 2015 Sep 14;10(9):e0137183. doi: 10.1371/journal.pone.0137183 (PMC4569069; doi:10.1371/journal.pone.0137183)
Supplement: S1 Protocol — (PDF) [file pone.0137183.s002.pdf]

## C1. Onderzoeksprotocol

# RESEARCH PROTOCOL

## September 2012

## PROTOCOL TITLE

Energy expenditure and sleep in response to protein/carbohydrate and fat ratio

| | |
|---|---|
| **Protocol ID** | NL39152 |
| **Short title** | Energy expenditure, sleep and macronutrients |
| **Version** | 4 |
| **Date** | February 2012 |
| **Coordinating investigator** | Prof. dr. M.S. Westerterp-Plantenga<br>Maastricht University, Department of Human Biology, Nutrition and Toxicology Research Institute Maastricht (NUTRIM)<br>P.O. Box 616, 6200 MD Maastricht<br>The Netherlands<br>E-mail: m.westerterp@maastrichtuniversity.nl<br>Phone: +31433881566<br>Fax: +31433670976 |
| **Principal investigator** | Dr. R. Hursel<br>Maastricht University, Department of Human Biology, Nutrition and Toxicology Research Institute Maastricht (NUTRIM)<br>P.O. Box 616, 6200 MD Maastricht<br>The Netherlands<br>E-mail: rick.hursel@maastrichtuniversity.nl<br>Phone: +31433882124<br>Fax: +31433670976<br><br>Msc. H.K.J. Gonnissen<br>Maastricht University, Department of Human Biology, Nutrition and Toxicology Research Institute Maastricht (NUTRIM)<br>P.O. Box 616, 6200 MD Maastricht<br>The Netherlands<br>E-mail: hkj.gonnissen@maastrichtuniversity.nl<br>Phone: +31433884596<br>Fax: +31433670976<br><br>Msc. E.A.P. Martens<br>Maastricht University, Department of Human Biology, Nutrition and Toxicology Research Institute Maastricht (NUTRIM)<br>P.O. Box 616, 6200 MD Maastricht<br>The Netherlands<br>E-mail: eap.martens@maastrichtuniversity.nl<br>Phone: +31433884596<br>Fax: +31433670976 |
| **Legal representative** | Nutrition and Toxicology Research Institute Maastricht (NUTRIM)<br>P.O. Box 616, 6200 MD Maastricht<br>The Netherlands<br>E-mail: mh.grispen@maastrichtuniversity.nl<br>Phone: +31433881476<br>Fax: +31433670286 |

| | |
|---|---|
| **Independent physician** | Prof. dr. W.H. Lamers<br>Maastricht University, Department of Anatomy & Embryology, Nutrition and Toxicology Research Institute Maastricht (NUTRIM)<br>P.O. Box 616, 6200 MD Maastricht<br>The Netherlands<br>E-mail: wh.lamers@maastrichtuniversity.nl<br>Phone: +31433881060<br>Fax: +31433884134 |
| **Laboratory sites** | Maastricht University |

**PROTOCOL SIGNATURE SHEET**

| **Name** | **Signature** | **Date** |
|---|---|---|
| **Legal representative:**<br>NUTRIM<br>Sef Janssen | | |
| **Coordinating Investigator**<br>Prof. dr. M.S. Westerterp-Plantenga | | |

**TABLE OF CONTENTS**

1. INTRODUCTION AND RATIONALE ..... 11
2. OBJECTIVES ..... 13
3. STUDY DESIGN ..... 14
4. STUDY POPULATION ..... 15
4.1 Population (base) ..... 16
4.2 Inclusion criteria ..... 17
4.3 Exclusion criteria ..... 17
4.4 Sample size calculation ..... 18
5. TREATMENT OF SUBJECTS ..... 19
5.1 Investigational product/treatment ..... 19
6. METHODS ..... 21
6.1 Study parameters/endpoints ..... 21
*6.1.1 Main study parameter/endpoint* ..... 21
*6.1.2 Secondary study parameters/endpoints* ..... 21
6.2 Randomisation, blinding and treatment allocation ..... 22
6.3 Study procedures ..... 22
*6.3.1 Screening* ..... 22
*6.3.2 Intervention* ..... 23
6.4 Withdrawal of individual subjects ..... 29
6.5 Replacement of individual subjects after withdrawal ..... 30
6.6 Follow-up of subjects withdrawn from treatment ..... 30
6.7 Premature termination of the study ..... 30
7. SAFETY REPORTING ..... 31
7.1 Section 10 WMO event ..... 31
7.2 Adverse and serious adverse events ..... 31
7.3 Follow-up of adverse events ..... 32
8. STATISTICAL ANALYSIS ..... 33
8.1 Descriptive statistics ..... 33
8.2 Univariate analysis ..... 33
8.3 Multivariate analysis ..... 33
9. ETHICAL CONSIDERATIONS ..... 34
9.1 Regulation statement ..... 34
9.2 Recruitment and consent ..... 34
9.3 Benefits and risks assessment, group relatedness ..... 34
9.4 Compensation for injury ..... 35
9.5 Incentives ..... 36
10. ADMINISTRATIVE ASPECTS AND PUBLICATION ..... 37
10.1 Handling and storage of data and documents ..... 37
10.2 Amendments ..... 37
10.3 Annual progress report ..... 37
10.4 End of study report ..... 38
10.5 Public disclosure and publication policy ..... 38

11. REFERENCES ........................................................................................................ 39

## LIST OF ABBREVIATIONS AND RELEVANT DEFINITIONS

| | |
|---|---|
| ABR | ABR form, General Assessment and Registration form, is the application form that is required for submission to the accredited Ethics Committee (In Dutch, ABR = Algemene Beoordeling en Registratie) |
| AEE | Activity-induced energy expenditure |
| ANOVA | Analysis of variance |
| AR | Adverse Reaction |
| BMI | Body Mass Index |
| BMR | Basal Metabolic Rate |
| CA | Competent Authority |
| CCMO | Central Committee on Research Involving Human Subjects; in Dutch: Centrale Commissie Mensgebonden Onderzoek |
| CV | Curriculum Vitae |
| DEE | Diet-induced energy expenditure |
| DER | Daily energy requirements |
| DSMB | Data Safety Monitoring Board |
| EB | Energy balance |
| ECG | Electrocardiography |
| EEG | Electroencephalogram |
| eGFR | Estimated Glomerular Filtration Rate |
| EMG | Electromyography |
| En% | Energy percent |
| EOG | Electrooculography |
| EU | European Union |
| FFA | Free fatty acids |
| FFM | Fat free mass |
| FM | Fat mass |
| FSR | Fractional synthetic rate |
| GCP | Good Clinical Practice |
| HCL | Hydrogen chloride |
| $^1$H-MRS | Proton magnetic resonance spectroscopy |
| IB | Investigator's Brochure |
| IC | Informed Consent |
| IMCL | Intramyocellular lipid |

| | |
|---|---|
| IMP | Investigational Medicinal Product |
| IMPD | Investigational Medicinal Product Dossier |
| MDRD | Modification of Diet in Renal Disease |
| METC | Medical research ethics committee (MREC); in Dutch: medisch ethische toetsing commissie (METC) |
| MPE | Mole percent excess |
| PAL | Physical activity level |
| POMS | Profile of Mood States |
| REM | Rapid eye movement sleep |
| RMR | Resting metabolic rate (consisting of SMR and DEE) |
| RQ | Respiratory quotient |
| (S)AE | (Serious) Adverse Event |
| SEM | Standard error of the mean |
| SMR | Sleeping metabolic rate |
| SPC | Summary of Product Characteristics (in Dutch: officiële productinfomatie IB1-tekst) |
| Sponsor | The sponsor is the party that commissions the organisation or performance of the research, for example a pharmaceutical company, academic hospital, scientific organisation or investigator. A party that provides funding for a study but does not commission it is not regarded as the sponsor, but referred to as a subsidising party. |
| SPSS | Statistical Package for the Social Sciences |
| STAI | State-trait anxiety inventory |
| SUSAR | Suspected Unexpected Serious Adverse Reaction |
| TEE | Total energy expenditure |
| TFEQ | Three Factor Eating Questionnaire |
| VAS | Visual analogue scale |
| WHR | Waist-to-hip ratio |
| Wbp | Personal Data Protection Act (in Dutch: Wet Bescherming Persoonsgevens) |
| WMO | Medical Research Involving Human Subjects Act (in Dutch: Wet Medisch-wetenschappelijk Onderzoek met Mensen |

## SUMMARY

**Rationale:** Protein has been observed to increase satiety and energy expenditure to a greater extent than carbohydrate and fat and can therefore reduce energy intake. However, it still has to be confirmed if this effect is permanent or transient over a longer period of time.

Moreover, dietary intakes may significantly affect sleep when macronutrient intakes are manipulated. Since sleep deprivation has been recognized as a risk factor for obesity, improving sleep by a change in macronutrient intake would be promising in the treatment of obesity.

**Objective**: To determine energy expenditure and sleep in response to protein/carbohydrate and fat ratio of the diet over a short-term and long-term period of time.

**Study design:** The study will be conducted in a parallel design. Allocation of subjects to the two conditions is randomized.

**Study population:** Forty healthy male and female subjects with a BMI between 18-27 kg/m$^2$ and aged between 18-35 years will be included in the study. All subjects will be non-smoking, weight stable, and at most moderate alcohol users without sleeping problems. Subjects will be free of medication except for oral contraceptive use in women.

**Intervention:** The subjects will stay three times 48 hours in the respiration chamber: at baseline, after 1 week (short-term effect) and after 12 weeks (long-term effect) of dietary intervention. After baseline measurement (protein intake of 15 energy percent), two conditions will be applied: protein intake of 5 and 30 energy percent. Fat content will be kept constant.

**Main study parameters/endpoints:** The primary endpoints of this study are energy expenditure, substrate oxidation and sleep. The secondary endpoints of this study are body composition, fat deposition and muscle protein synthesis rate, expressed as fractional synthetic rate (FSR).

**Nature and extent of the burden and risks associated with participation:**

This study does not include major risk factors for the subjects. Blood sampling in this study is limited and without side effects, except from a minor risk of bruising. Deuterium is an isotope of water that naturally appears in the body. Drinking it does not expose the subject to any risks. Urine sampling will be done in urine bottles added with diluted HCl, which might pose a risk for the subjects. However, subjects will be carefully instructed on how to handle the bottles to reduce these risks. MRI and MRS are safe, non-invasive procedures with no ionizing radiation and these procedures do not expose the subjects to any risks if the exclusion criteria (no electronic implants, no pacemakers, no metal fragments in the eyes, skin or body) are well respected. Additionally, there are no risks for the subject in consuming any of the provided meals, because people with certain food allergies are excluded for

participation and all food items will be commercially available in normal Dutch supermarkets. A 30En% protein or a 5En% protein diet for 12 weeks will not have any side effects on the subject's health. Studies in the respiratory chamber will be conducted using standard operating procedures. A pair of subjects will always participate in the study at the same time and therefore they will never be alone. The subjects will be able to contact the investigators during the entire night. In addition, they will be able to get out of the chamber at any time they feel uncomfortable.

For measuring muscle protein synthesis (additional, voluntary test day) rate two catheters will be inserted in a forearm vein and four muscle biopsies will be obtained. Insertion of the catheters is comparable to a normal blood draw and the only risk is a small local hematoma. This is the same for the muscle biopsies. Muscle biopsies will be taken through a small (5 mm) incision, following local anesthetics of the skin and muscle fascia, and will heal completely. Muscle biopsies will only be obtained by an experienced physician. The test beverages contain intrinsically labeled dietary protein which is safe for human consumption and have been used in previous studies (MEC 06-3-064, MEC 07-3-086, MEC 09-3-078.3). The labeled, non-radioactive amino acids tracers that will be infused intravenously are produced according to GMP standards and are completely safe. The experiment will take about 162 hours of the subject's time.

**Benefit:** This study does not have any benefits for the subjects themselves, but will give possible new knowledge for the treatment of obesity.

# 1. INTRODUCTION AND RATIONALE

Obesity is associated with an increased risk for chronic diseases including type 2 diabetes, cancer, and cardiovascular diseases (2). The development of obesity results from a chronic energy imbalance, with energy intake exceeding energy expenditure. Both decreasing energy intake and increasing energy expenditure may result in weight loss. Modest weight loss (5–10 percent of body weight) is associated with clinical improvements in insulin sensitivity and fasting glucose levels, thereby reducing the risk for the development of type 2 diabetes (3). Furthermore, reductions in dyslipidemia and hypertension as a result of weight loss reduce the risks for cardiovascular diseases (4).

Weight loss strategies regarding food intake regulation mainly focused on changing patterns of fat and carbohydrate consumption during the last decades (5, 6). The role of protein has largely been ignored, because it typically comprises only about 15 percent of daily energy intake, and is near constant within and across populations (7). However, with respect to energy expenditure, it is known that protein has the highest and the most prolonged effect of the separate macronutrients (8). Protein has been observed to increase satiety to a greater extent than carbohydrate and fat and can therefore reduce energy intake (9, 10). High protein diets were previously shown to increase energy expenditure in healthy subjects (11, 12). A high protein diet (30En% of protein), compared with an adequate-protein diet (10En%), fed at energy balance for 4 days increased 24h satiety, thermogenesis, sleeping metabolic rate, protein balance and fat oxidation (11). However, the effect of a low protein diet (5En%) on energy expenditure and substrate oxidation has been never looked at before. Moreover, it remains unclear whether the effect of protein on energy expenditure is a permanent or transient effect when you maintain a high protein diet for 12 weeks. Increased basal (muscle) protein synthesis, after a high protein diet, may be one of the factors that explain an increase in energy expenditure. This increase in energy expenditure may be absent during the low protein diet because of protein breakdown rather than protein synthesis. On the other hand, post-prandial protein synthesis may be higher in the low protein group compared to the high protein group because subjects become more sensitive for protein and utilize the consumed amino acids more efficient.

The average sleep duration and sleep quality decreased together with an increasing prevalence of obesity. People, who sleep less than 7 hours a night, are at higher risk to become obese (13). Sleep restriction may effect energy balance and result in an up-regulation of appetite, more time to eat and a decrease of energy expenditure, probably because hormone levels change due to lack of sleep (14, 15). In addition, it has been shown

that sleep plays an important role in the preservation of human fat-free body mass (FFM) during periods of caloric restriction. Nedeltcheva et al. 2010 reported that the combination of energy and sleep restriction in overweight adults resulted in a modified state of negative energy balance characterized by decreased loss of fat and considerably increased loss of fat-free body mass (16).

Recently, it has been shown that dietary intakes may significantly affect sleep, measured by Actigraph sleep watches, when macronutrient intakes are manipulated. High-protein diets were associated with significantly fewer wake episodes and high-carbohydrate diets were associated with significantly shorter sleep latencies than control diets (17). Feeding-induced sleep modulation probably results from an alternation of the availability of various neurotransmitters. Among food components, amino acids serve as precursors for the synthesis of different neurotransmitters. Trypthophan is such an amino acid, which serves as a precursor for serotonin. Serotonin, in turn is converted into melatonin. Through its ability to elevate brain serotonin and melatonin levels, trypthophan may have the potential to improve sleep disturbances resulting from nutritional deficiencies or environmental disturbances (1, 18). Moreover, increased protein intake has shown to result in greater loss of fat mass and less loss of fat free mass during energy restriction, and lower regain of fat mass and greater regain of fat free mass during weight regain after weight loss (19-21). Although increased protein intake has been shown to decrease body fat content, the effect of protein intake on fat distribution remains unknown. Fat distribution, especially ectopic fat deposition, has been suggested as an important determinant of insulin sensitivity and has to be considered as part of body fat content (22). Visceral and ectopic fat distribution, as well as regional muscle mass can be estimated via readily available imaging techniques such as MRI and MRS (23).

The present study aims to determine energy expenditure, substrate oxidation and muscle protein synthesis rates with diets contrasting in protein content over a longer period of time. By determining the long-term effect of high protein and low protein diets it will be possible to elucidate if the effect of protein on energy expenditure is a permanent or transient effect, as well as the contribution of protein synthesis to the protein induced thermogenesis. Moreover, the study aims to determine the different effects of diets contrasting in protein content on sleep duration and sleep quality, measured by electroencephalography. We hypothesize that the diet with 30En% of protein will affect sleep, energy expenditure and will have a fat free mass sparing effect by increasing protein synthesis. The diet with 5En% of protein will not affect sleep, energy expenditure and will not spare fat free mass. By comparing subjects with different sleep duration and quality, it will be possible to confirm the fat free mass sparing effect of sleep.

As a secondary aim the present study determines body composition (including body fat content, fat mass and fat free mass), body fat distribution (including abdominal, liver and muscle fat) and (muscle) protein synthesis in response to diets contrasting in protein content.

## 2. OBJECTIVES

Primary Objectives:

To determine energy expenditure and substrate oxidation in response to protein/carbohydrate and fat ratio of the diet

To determine sleep duration and sleep quality in response to protein/carbohydrate and fat ratio of the diet.

Secondary Objectives:

To determine body composition in response to protein/carbohydrate and fat ratio of the diet

To determine body fat distribution in response to protein/carbohydrate and fat ratio of the diet

To determine (muscle) protein synthesis and regional muscle mass in response to protein/carbohydrate and fat ratio of the diet

## 3. STUDY DESIGN

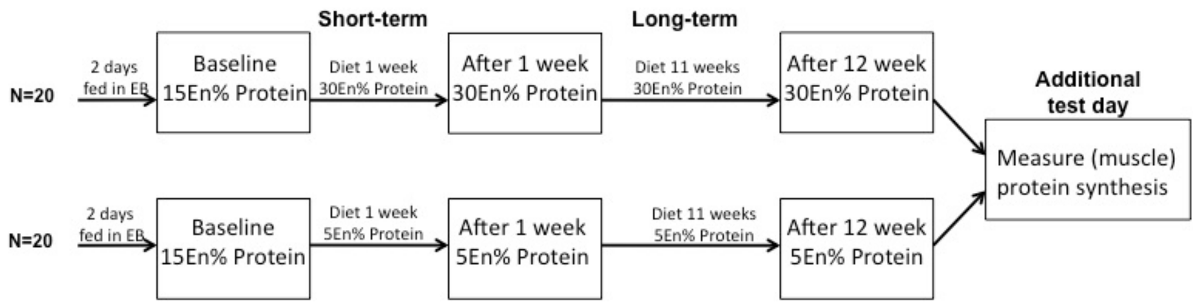


**Figure 1**: Flow diagram of study design.

The study will have a parallel design and will consist of a short-term (1 week) and long-term (12 weeks) dietary intervention period.

Two days before the baseline measurement, subjects will be fed in energy balance (EB) with protein intake of 15En%. After the baseline measurement, subjects (n=40) will be randomly divided in two groups (2 x n=20). The two applied conditions will differ in the absolute protein content of the meals, including 5En% and 30En% from protein. Fat content will be kept constant in all three conditions regarding the possible effects of fat intake on fat oxidation, resulting in macronutrient compositions of the diets of En% Protein/Carbohydrate/Fat: 5/60/35 and 30/35/35. Both conditions will be compared with the baseline measurement when subjects are fed in energy balance with a macronutrient composition of 15/50/35 En% (Protein/ Carbohydrate/Fat). During the 12 weeks dietary intervention period, subjects will receive recipes prescribed by a qualified dietician. The 30En% protein group will receive recipes for high-protein meals and in addition protein supplements. The 5En% protein group will receive recipes for low-proteins meals and in addition carbohydrate supplements.

To determine energy expenditure and substrate oxidation, subjects will stay in the respiration chamber for 48 hours at baseline, after 1 week and after 12 weeks of an energy balanced diet. During the stay in the chamber, polysomnography will be used to measure wake and sleep phases continuously, in order to determine sleep duration and sleep quality. Every morning blood samples will be taken in fasted state to determine plasma concentrations of glucose, insulin and free fatty acids. Appetite and mood profiles will be measured hourly and before and after each meal, by anchored 100mm visual analogue scales and by POMS and STAI-state questionnaires.

At the start and after 12 weeks, 3-compartment body composition will be determined with the deuterium dilution technique combined with densitometry (BodPod). To investigate fat distribution MRI and $^{+}$H-MRS will be used. Abdominal fat will be assessed at baseline, and liver fat and intramyocellular lipid (IMCL) content, as well as the mass of the vastus lateralis muscle will be measured at baseline and after 12 weeks.

To assess dietary protein intake and thus compliance to the diet, subjects will complete 24-hour urine collections at the start, during their stays in the respiration chamber and at week 11. One week before the baseline measurement and at week 11 an accelerometer will be worn to measure habitual physical activity.

During the screening, after 6 weeks and after 12 weeks of high protein diet the glomerular filtration rate (GFR) will be estimated using the Modification of Diet in Renal Disease (MDRD) study equation (for detail see section 6.3).

After the final measurement in the respiration chamber the subjects are invited for a final test day in which (muscle) protein synthesis will be measured. This additional test day is not compulsory and subjects that do not want to participate can still take part in the rest of the study.

During the test day, subjects will receive a test-drink containing 20 g intrinsically labelled casein to be able to measure the effect of protein ingestion on muscle protein synthesis. This allows us to determine the true incorporation of amino acids from the ingested, intact casein into the muscle, taken into account the influence of digestion and absorption processes of a whole protein (24-27). Intrinsically labelled milk proteins were derived from milk that has been collected from cows that were infused with L-[1-$^{13}$C] phenylalanine (27). This procedure has been successfully used in human intervention studies by the research group of Prof. Dr. L. van Loon (MEC 06-3-064, MEC 07-3-086, MEC 09-3-078.3, MEC 10-3-080), which has already resulted in multiple publications (24-27). Subsequently, plasma and muscle samples will be collected. Together with the use of an intravenous L-[ring-$^{2}H_{5}$]phenylalanine, L-[1-$^{13}$C]-leucine and L-[ring-$^{2}H_{2}$]tyrosine infusion, the digestion and absorption kinetics of the ingested protein, whole body protein balance and the fractional synthetic rate (FSR) of mixed muscle protein in the fasted and fed state in an *in vivo* human setting will be assessed.

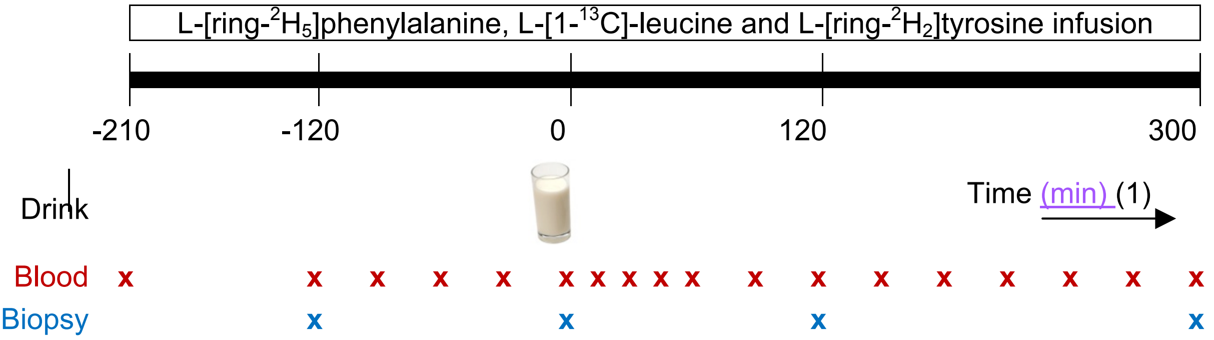


**Figure 2**: time schedule of the additional test day.

# 4. STUDY POPULATION

## 4.1 Population (base)

The study population will consist of 40 healthy, non-smoking, normal weight subjects (20 subjects within each condition) matched in BMI, age and gender. Recruitment will take place by advertising around the campus and in local newspapers.

Interested candidates will be sent the information brochure of the study, including the main inclusion criteria. In case the potential subject wants to participate and meets the main inclusion criteria, an appointment will be set for a screening visit.

During the screening:

- An informed consent form needs to be signed by the subject.
- Determination of weight and height, calculation of the Body Mass Index (BMI).
- Determination of waist and hip circumferences.
- One blood sample of 10 mL per subject will be drawn to determine plasma creatinine concentrations in order to estimate the GFR
- Questionnaires will be completed to assess
    - Medical situation at the present time and in the past. An example of this questionnaire is attached to the protocol.
    - Sleeping behaviour. An example of this questionnaire is attached to the protocol.
    - Eating pattern by the Dutch translation of Three Factors Eating Questionnaire (28). This questionnaire consists of three subscales: cognitive restraint of eating, disinhibition of dietary restraint and emotional eating, and perceived hunger. An example of this questionnaire is attached to the protocol.
    - Habitual physical activity by a Dutch translation of the Baecke questionnaire (29). This questionnaire consists of three subscales: work activity, sports activity and non-sports leisure activity. This questionnaire is included in the questionnaire about the medical situation of the subjects attached to the protocol.

The results of the completed questionnaires will be checked on the inclusion and exclusion criteria before assigning the subject to the study population.

### 4.2 Inclusion criteria

- Age between 18 and 35 years
- BMI between 18 and 27 kg/m2
- Non-smoking
- No sleeping problems
- Normal general health (screening questionnaire)
- No medication that could interfere with the experiment
- Unrestrained eaters (F1 < 9)
- Weight stable (weight change < 3 kg during the last 6 months)
- eGFR ≥ 90ml/min/1.73m$^2$

### 4.3 Exclusion criteria

- Age under 18 and over 35 years
- BMI under 20 and over 27 kg/m2
- Smoking
- Sleep problems (such as insomnia, daytime hypersomnia or repeated waking up during the night)
- Not in good health
- Medication use that could interfere with the experiment
- Food allergies
- Dietary restrained eaters (F1 > 9)
- Excessive physical activity
- Consumption of more than 10 alcoholic drinks per week
- Change in body weight of more > 5 kg in the last 6 months prior to study entry
- eGFR < 90ml/min/1.73m$^2$
- Exclusion criteria for MRI (electronic implants, pacemakers, metal fragments in the eyes, skin or body)
- Use of anticoagulants, blood diseases, allergy for lidocain
- Use of gastric acid inhibitors
- All co-morbidities interacting with mobility and muscle metabolism of the lower limbs (e.g. arthrosis, arthritis, spasticity/rigidity, all neurological disorders and paralysis).

### 4.4 Sample size calculation

To do a sample size calculation a respiration chamber study on the effect of two diets with different macronutrient compositions on energy expenditure was used.

Westerterp *et al.1999* report Diet-induced-Thermogenesis of 14.6 ± 2.9 % and 10.5 ± 3.8 % when changing from 29% of energy from protein to 9% of energy from protein in the diet (12). Sample size was calculated using G*Power version 3.2.1. With the group mean Diet-induced-Thermogenesis change from the past research (12), G*Power first calculates the effect size. The calculated effect size was 1.212986. With an $\alpha$ of 0.05 and $\beta$ of 0.10 (power=1-$\beta$=0.90) and an effect size of 1.212986, the program calculates that we need 16 subjects per group.

Taking into account a drop out rate of ±20%, 20 subjects per condition will be included.

When the subject leaves the respiration chamber before the end of the measurement, this subject is considered as a drop-out.

# 5. TREATMENT OF SUBJECTS

## 5.1 Investigational product/treatment

The 12 weeks dietary intervention period used in this study includes two diets differing in protein content (5En% and 30En%). The 30En% protein group will receive recipes for high-protein meals and in addition protein supplements. The 5En% protein group will receive recipes for low-proteins meals and in addition carbohydrate supplements. The protein and carbohydrate supplements are easily solved in water and can be consumed as shakes.

An example of a high and a low protein menu.

| | **30En% protein** | **5En% protein** |
|---|---|---|
| Breakfast | cereals<br>semi-skimmed milk | cereals<br>breakfast drink (juice-based drink) |
| Shake | whey-protein (40g)<br>solved in water | fantomalt (40g)<br>solved in water |
| Lunch | bread<br>cheese | bread<br>cucumbersalade |
| Snack | skimmed yoghurt | syrup waffle |
| Shake | whey-protein (40g)<br>solved in water | fantomalt (40g)<br>solved in water |
| Dinner | nasi goreng<br>chicken<br>vegetables | nasi goreng<br>peanut butter sauce<br>vegetables |

The recommended protein-rich and protein-poor food items will be commercially available in normal Dutch supermarkets.

The chosen protein supplement is an $\alpha$-lactalbumine rich whey protein. This protein supplement is chosen because $\alpha$-lactalbumine has been shown to increase the ratio of plasma tryptophan to the sum of other amino acids (30). Through its ability to elevate brain serotonin and melatonin levels, tryptophan may have the potential to improve sleep disturbances (as described in the introduction).

## 5.2 Test beverage additional test day

Protein intake is important to stimulate muscle protein synthesis. During the test day, intact casein will be investigated since casein is one of the main proteins that is naturally present in normal milk and it is a representative protein. The same intact casein that has been used previously (MEC 07-3-086, MEC 09-3-078, MEC 10-3-080), will be used again in this study. All subjects will receive a beverage of a volume of 400 mL water containing intact casein. L-

[1-13C]phenylalanine labelled milk proteins are produced in collaboration with Dr. Boirie (Clermont-Ferrand, France) using registered cattle for milk production applying quality process #0000640 in accordance with standard NF V 01-005. Milk proteins were processed to obtain purified casein by the NIZO food research (Nederlands Instituut voor ZuivelOnderzoek, Ede, the Netherlands). During the processing of the milk, total plate count and several tests for micro-organisms were performed (*B. cereus*, *S. aureus*, Coliforms, moulds yeast, Salmonellae and Lysteria) to assure that no contamination occurred during processing. The intact casein was tested according to NIZO's manufacturing specifications conform the performance typical for this grade and product description, before clearance was given for use in human subjects (Appendix D2.1). The casein was stored at -20 ºC in the "dietary-kitchen" at the department of Human Biology. The beverage will be uniformly flavoured by adding 5 g of cream vanilla flavour for each litre of beverage (Givaudan, Naarden, the Netherlands (Appendix D2.2)). Drinks will be prepared according to Standard Operating Procedure #RM001, as routinely applied in the kitchen of the department of Human Biology the morning before the start of the test day.

### 5.2.1 Intravenous tracer infusion

The stable isotopic tracers (L-[$^2H_5$]-phenylalanine, [$^2H_2$]-tyrosine and L-[1-$^{13}$C]-leucine) will be purchased from Cambridge Isotopes (Andover, USA) and dissolved in 0.9% saline before infusion. Tracer solutions for intravenous administration will be prepared by Basic Pharma following GMP-standards (Appendix D2.3). Continuous intravenous infusion during the entire period of the test day (8½ h, 0.05 µmol/kg/min L-[$^2H_5$]-phenylalanine, 0.02 µmol/kg/min [$^2H_2$]-tyrosine, 0.12 µmol/kg/min L-[1-$^{13}$C]-leucine) of the labeled stable isotopes will be performed using a calibrated IVAC 560 pump (San Diego, USA). The phenylalanine, tyrosine and leucine pools will be primed, such that whole body steady state labeling of the amino acids will be achieved within 90 min. Using these tracers in whole body phenylalanine kinetics can be calculated using established tracer models (31), which have been applied in multiple previous studies within our group (24-26, 32-38).

# 6. METHODS

## 6.1 Study parameters/endpoints

### *6.1.1 Main study parameter/endpoint*

The main study parameters are energy expenditure, substrate oxidation and sleep.

- energy expenditure (MJ/day), which will be measured via oxygen consumption and carbon dioxide production in the respiration chamber
- substrate oxidation (gram/day); carbohydrate, fat and protein oxidation will be calculated using oxygen consumption, carbon dioxide production and urinary nitrogen excretion, with the formule of Brouwer (39). Urine will be collected during the stay in the respiration chamber. Nitrogen concentration will be measured using a nitrogen analyser.
- sleep (minutes) will be registered continuously with polysomnography

### *6.1.2 Secondary study parameters/endpoints*

- body composition (% fat, kg FM and kg FFM), which will be calculated from body volume and total body water using the combined equation of Siri (40). Body volume will be measured with a BodPod. Total body water will be measured with Deuterium dilution according to the Maastricht protocol (41).
- fat distribution (abdominal fat, liver fat and IMCL), which will be determined with MRI and $^1$H-MRS
- Regional muscle mass of the upper part of the lower limbs, which will be determined with MRI
- plasma concentrations of glucose (mmol/L), insulin (mU/L) and free fatty acids (FFA, mmol/L) which will be measured before each breakfast in the respiration chamber by drawing one blood sample of 10ml from the antecubital vein of the contra-lateral arm using venapunction
- physical activity (counts/min) one week before baseline measurement and one week before final measurement, which will be measured using a triaxial accelerometer for movement registration (Tracmor$_D$; DirectLife, Philips new wellness solutions, Amsterdam, The Netherlands) sensitive to a wide range of body movements.
- mood, feeling of hunger and satiety, which will be measured hourly and before and after each meal, by Visual Analogue Scales (VAS) and by Profile of Mood States (POMS) and State Trait Anxiety Index (STAI) questionnaires (42, 43).
- 6-sulfatoxymelatonin (urinary metabolite of melatonin, ng/day) and 5-hyrdoxy-3-indole acetic acid (urinary metabolite of serotonin, ng/day) will be measured
- muscle protein synthesis rate, expressed as fractional synthetic rate (FSR):

In order to determine the FSR, the following parameters will be measured in blood and muscle tissue:

- Plasma free phenylalanine and leucine concentration (expressed as μmol/L)
- Plasma enrichment of L-[ring-$^{2}H_{5}$]phenylalanine, L-[1-$^{13}C$]phenylalanine and L-[1-$^{13}C$]-leucine (expressed as mole percent excess (MPE))
- Muscle free and protein bound enrichment of L-[1-$^{13}C$]phenylalanine, L-[1-$^{13}C$]-leucine and L-[ ring-$^{2}H_{5}$]phenylalanine (expressed as MPE)

**-** whole body protein turnover:Whole body protein turnover consist of the following rates:

- Exogenous phenylalanine rate of appearance and plasma availability of phenylalanine.
- Total rate of phenylalanine appearance and disappearance (= protein turnover)
- Endogenous phenylalanine rate of appearance (=protein breakdown)

In order to determine these rates, the following parameters will be measured in blood:

- Plasma free phenylalanine, tyrosine and leucine concentration (expressed as μmol/L)
- Plasma enrichment of L-[ring-$^{2}H_{5}$]phenylalanine, L-[ring-$^{2}H_{2}$]tyrosine, L-[1-$^{13}C$]phenylalanine, and L-[1-$^{13}C$]-leucine (expressed as mole percent excess (MPE))

## 6.2 Randomisation, blinding and treatment allocation

The study will be conducted in a double-blinded parallel design. The allocation of subjects to the groups with protein intake of 5 or 30 energy percent is randomized. Randomisation will take place by the dietician using a computerized randomization programm ('Randomizer' macro in Excel).

## 6.3 Study procedures

### *6.3.1 Screening*

*Anthropometrics*

Body weight will be measured using a digital balance with subjects in underwear, in the fasted state and after voiding their bladder. Height will be measured by a wall-mounted stadiometer. BMI will be calculated as body weight (kg) divided by height (m) squared. Waist and hip circumference will be determined in standing position by a tape measure. Waist circumference will be measured at the smallest circumference between rib cage and iliac crest, and hip circumference at the level of the spina iliaca anterior superior. Accordingly, waist-to-hip ratio (WHR) will be calculated by dividing waist circumference by hip circumference. Both waist circumference and WHR will be used to define different patterns of body fat distribution.

*Eating behaviour*

Eating behavior will be analyzed using a Dutch translation of the Three Factor Eating Questionnaire (TFEQ), which measures the three factors (F) involved in eating behavior, namely ‘cognitive restraint of eating’ (F1), ‘disinhibition of restraint’ (F2) and ‘hunger’ (F3) (28). Dietary restraint reflects the extent to which individuals attempt to cognitively control their food intake. Disinhibition reflects the extent to which release from the cognitive suppression of eating occurs in response to the presence of palatable food or other disinhibiting stimuli, such as emotional distress. Hunger refers to the subjective feeling of hunger. Based upon the median in the population from south of the Netherlands, dietary restraint was defined by TFEQ F1 scores ≥ 9. F1 scores < 9 indicated dietary unrestraint (14). High disinhibition was defined by TFEQ F2 scores ≥ 5, and F2 scores < 5 represented low disinhibition. Completing the questionnaire will take about 3 minutes.

*Anxiety*

The STAI state questionnaire refers to the transitory emotional response involving unpleasant feelings of tension and apprehensive thoughts. The STAI scale is composed of 20 questions rated on a four-point scale, ranging from ‘much like this’ to ‘much unlike this’ and requires that subjects describe how they feel generally (Trait Anxiety Inventory scale) The questionnaire can score a maximum of 80, whereby increased scores are associated with an increase in anxiety (43). Completing both questionnaires will take about 3 minutes.

*Physical activity*

Habitual physical activity is evaluated using a Dutch translation of the Baecke questionnaire. This questionnaire consists of three subscales: work activity, sports activity and non-sports leisure activity (29).

*Glomerular Filtration Rate*

The glomerular filtration rate will be estimated using the MDRD study equation. The MDRD equation uses four variables (serum creatinin, age, race and gender) to estimate the glomerular filtration rate (44, 45). To determine serum creatinin concentrations, a blood sample of 10ml will be collected via vena puncture in the antecubital vein.

### 6.3.2 Intervention

*Energy expenditure*

Oxygen consumption and carbon dioxide production will be measured in the respiration chamber. The respiration chamber is a 14m3 hotel room, furnished with a bed, chair, computer, television, radio, dvd-player, telephone, intercom, sink and toilet. The room is

ventilated with fresh air at a rate of 70-80 l/min. The ventilation rate is measured with a dry gas meter. The concentrations of oxygen and carbon dioxide will be measured using a paramagnetic O2 analyzer and an infrared CO2 analyzer. During each 15-min period six samples of outgoing air for each chamber, and one sample of fresh air, zero gas, and calibration gas will be measured. The gas samples to be measured will be selected by a computer that also stores and processes the data (46).

24-h energy expenditure consists of sleeping metabolic rate (SMR), diet-induced energy expenditure (DEE), and activity-induced energy expenditure (AEE). 24-h energy expenditure and 24-h respiratory quotient (RQ) will be calculated using the formula of Brouwer (39). Physical activity is monitored using a radar system working on the Doppler principle. SMR is defined as the lowest mean energy expenditure measured over three consecutive hours between 00:00h and 07:00h. DEE will be calculated by plotting energy expenditure against radar output, both averaged over 30-min periods. The intercept of the regression line at the lowest radar output represents the energy expenditure in the inactive state (Resting Metabolic Rate; RMR), consisting of SMR and DEE (47). DEE will be determined by subtracting SMR from RMR. Activity-induced energy expenditure will be determined by subtracting RMR from 24-h energy expenditure. The Physical Activity Level (PAL) will be calculated by dividing 24-h energy expenditure by SMR.

*Substrate oxidation*

Carbohydrate, fat and protein oxidation will be calculated using oxygen consumption, carbon dioxide production and urinary nitrogen excretion, with the formule of Brouwer (39). Urine will be collected during each 48h stay in the respiration chamber. Samples will be collected in containers with 10ml diluted HCl to prevent nitrogen loss through evaporation. The subjects will be told, before entering the respiration chamber, to handle the urine containers with the most caution because of the presence of the diluted HCl. Volume and nitrogen concentration will be measured, the latter using a nitrogen analyser.

*Sleep*

During each stay in the respiration chamber, sleep will be monitored continuously via polysomnography. The subject is only allowed to sleep on the times stated by the investigator, so daytime naps and sleeping is not allowed, and will be controlled via the polysomnographic measurements. Polysomnography typically consists of 4 measurements: EEG, EMG, EOG and ECG. To measure brain activity (EEG) 6 electrodes are placed on the head. These electrodes provide a readout of the brain activity that can be "scored" into different stages of sleep (N1, N2, N3 which combined are referred to as NREM sleep, and rapid eye movement sleep or REM, and Wakefulness). In addition, to determine if sleep

occurs and especially to determine when REM sleep occurs, 2 electrodes on the chin and 2 electrodes on the eyes are necessary to measure muscle activity (EMG) and eye movements (EOG). Finally, 2 additional electrodes are placed on the chest to monitor heart rate and cardiac function (ECG). The combination of these data will make it possible to determine sleep duration and sleep quality. Sleep quality represents the amount of minutes in which the subject is in sleep stage N3 plus REM sleep divided by the total duration of sleep.
Subjective feelings of sleep will be assessed before each meal in the respiration chamber using 100 mm VAS with questions on subjective feelings of napping, physical exhaustion, alertness, sleepiness and how satisfying the sleep was. Opposing extremes of each feeling are described at either end of the 100-mm horizontal line, and subjects mark the line to indicate how they feel at that moment. An example of this questionnaire is attached to the protocol.

*Body composition*

Subjects' body composition will be calculated from body volume and total body water using the combined equation of Siri (40). Body volume is measured with a BodPod. Total body water is measured with Deuterium dilution according to the Maastricht protocol (41). Subjects drink a Deuterium dilution (about 70 g with an enrichment of 5 atom% excess $^{2}H$) in the evening prior to the measurements after the collection of a baseline urine sample. The next morning after a 10-hour equilibration period, where subjects are not allowed to eat or drink, a second urine sample is collected.

*Magnetic resonance imaging (MRI) and Magnetic resonance Spectroscopy (MRS)*

MRI and MRS are non-invasive and non-irradiant imaging techniques, which will be used to determine abdominal fat and muscle mass of the upper part of the lower limbs (MRI), muscle and liver fat (MRS).
An MRI (magnetic resonance imaging) scan will be performed with a whole body scanner (Intera, Philips Medical Systems) to quantify fat distribution and regional muscle mass. A single-slice breath-hold turbo spin-echo (TSE) sequence (turbo factor 5, echo time TE 12 ms, TR 200 ms, slice thickness 10 mm, field-of-view (FOV) 420-450 mm, total measurement time 11 s) is modified to suppress the water signal so that the images contain mainly the fat signal. The breath-hold technique is used to avoid any motion artifacts (ghosting due to the movement of subcutaneous fat regions with high signal intensity), which would cause difficulties during the data segmentation. This technique is performed to measure fat content in the subcutaneous abdominal and visceral fat. Detailed measurement of abdominal fat was described by Tintera et al. (48). Transversal images will be made from the upper part of the lower limbs. Muscle mass will be expressed as volumes (cubic centimeters), derived

by using tissue area measurements from multiple images. MRI is a safe procedure with no ionizing radiation and no known risks as long as the exclusion criteria (no electronic implants, no pacemakers, no metal fragments in the eyes, skin or body) are not met.
Image guided localized single voxle 1H-MRS will be performed in the m.vastus lateralis at the start and after 12 weeks. The measurements are performed on a whole body scanner (Intera, Philips Medical System, Best, the Netherlands) with a flexible surface coil wrapped around the upper leg. This technique is performed to measure fat content in the muscle (intramyocellular lipid; IMCL). Detailed intramycellular lipid measurement is described by Schrauwen-Hinderling et al. (49). While fat content in the liver will be determined using an MRS protocol as described by Stefan et al. (50).

*Blood samples*

Plasma concentrations of glucose, insulin and which will be measured before each breakfast in the respiration chamber by drawing one blood sample of 10ml from the antecubital vein of the contra-lateral arm using venapunction. Within this study, a total of 174 mL venous blood will be collected from each subject, i.e. 3 x 10 mL for the glucose and insulin and 144 mL (18x8mL) during the additional test day. Aliquots of plasma will be frozen in liquid nitrogen and stored at –80°C until analysis of glucose (Uni Kit III, La Roche, Basel, Switzerland) and insulin by radio immunoassay (Linco, Human Insulin RIA kit). Phenylalanine, leucine and tyrosine enrichment in plasma samples and in the muscle free amino acid pool will be measured using gas chromatography-mass spectrometry (Agilent G1530N/G2589A, Little Falls, USA), while the phenylalanine enrichment in muscle proteins will be measured with GC-combustion IRMS (Finnigan MAT 252, Bremen, Germany) (51). In all plasma samples glucose, insulin and amino acid concentrations and amino acid tracer/tracee ratio's will be measured.

*Physical activity.*

Habitual physical activity will be measured using a triaxial accelerometer for movement registration (Tracmor$_D$; DirectLife, Philips new wellness solutions, Amsterdam, The Netherlands) sensitive to a wide range of body movements. The accelerometer has been validated with doubly labelled water, the gold standard for measuring energy expenditure in daily life (52). The accelerometer will be worn under free-living conditions one week before the baseline measurement and one week before the final measurement. Subjects will be instructed to wear the Tracmor$_D$ from the moment they wake up in the morning until they go back to bed at night. The Tracmor$_D$ is only a small device that will be worn on a belt around

the waist. To verify whether subjects live up to this instruction, waking hours and clock times of wearing the $Tracmor_D$ have to be noted.

*Mood, feelings of hunger and satiety*

Appetite and mood profiles will be measured hourly and before and after each meal, by visual analogue scales (VAS) and by Profile of Mood States (POMS) and State Trait Anxiety Index (STAI) questionnaires (42, 43). Examples of these questionnaires are attached to the protocol.

Aspects of appetite will be assessed using 100 mm VAS with questions about feelings of hunger, satiety, thirst, and desire to eat. Opposing extremes of each feeling are described at either end of the 100-mm horizontal line, and subjects mark the line to indicate how they feel at that moment.

The POMS questionnaire contains 70 adjectives that are rated on a five-point scale, anchored by “much like this” to “much unlike this” and is divided into five subscales (depression, tension, confusion, fatigue and anger), each scoring a maximum of 35. An increase in POMS scores is associated with a worsening in mood, except in the case of “vigor”.

The STAI state questionnaire refers to the transitory emotional response involving unpleasant feelings of tension and apprehensive thoughts. The STAI scale is composed of 20 questions rated on a four-point scale, ranging from “much like this,” to “much unlike this” and requires that subjects describe how they feel generally, on the anxiety-trait scale, and how they feel at a specific moment, on the anxiety-state scale. The questionnaire can score a maximum of 80 and an increase in STAI state scores is associated with an increase in anxiety.

Filling out the questionnaires will take about a minute per questionnaire, in total 3 minutes.

*Melatonin and serotonin*

Urine will be collected during each 48h stay in the respiration chamber to measure 6-sulfatoxymelatonin (urinary metabolite of melatonin) and 5-hyrdoxy-3-indole acetic acid (urinary metabolite of serotonin). An enzyme-linked immunosorbent assay will be used to determine urinary 6-sulfatoxymelatonin and 5-hyrdoxy-3-indole acetic acid levels.

*Energy intake*

Our subjects are in overall energy balance, as they are weight stable for at least three months and not currently on a diet. Two days before the first stay in the respiration chamber and during all their stays in the respiration chambers, subjects will be fed in energy balance, Calculations will be based upon average daily energy requirements (DER). The daily energy

requirement for the two days before the experiment will be estimated as 1.75 times the Basal Metabolic Rate (BMR) (53). BMR will be calculated with the equation of Harris and Benedict (54). The energy requirement in the respiration chamber will be calculated as 1.4 times the measured sleeping metabolic rate (SMR) of the first night. The selected activity index is based on results of a study in which physical activity was determined in confined conditions (a respiration chamber) resulting in a mean PAL of 1.4 (calculated as 24-h energy expenditure/SMR) (55). Daily energy intake will be divided over three meals: breakfast 20%, lunch 40% and dinner 40%. Breakfast will be given at 08:30 hours, lunch at 13:30 hours and dinner at 18:30 hours. During the first stay in the respiration chamber, the macronutrient composition of the diet will be 15/50/35 En% (Protein/ Carbohydrate/Fat). During the second and third stay in the respiration chamber, the macronutrient composition of the diet will be 5/60/35 En% for the one group and 30/35/35 En% for the other group. The diets consist of normal food products, which are commercially available in normal Dutch supermarkets. The protein supplements for the diet with 30 En% of protein and the carbohydrate supplements for the diet with 5 En% of protein are commercially available. A qualified dietician will freshly prepare the meals in a qualified research kitchen, just before they are offered to the subjects.

The use of polysomnography and MRI are exclusively intended for scientific purposes. The researchers are not trained to make a clinical diagnosis of the results. However, it could be that by coincidence a possible deviation may be found. In this case, a medical expert will look at the data and when necessary the subject will be referred to a medical specialist.

*(Muscle) protein synthesis*

An outline of the study protocol of a test day is shown in **Figure 2**. At 8.00 am, following an overnight fast, subjects will arrive at the laboratory by car or public transportation. A Teflon catheter (Baxter BV, Utrecht, the Netherlands) will be inserted into an antecubital vein for intravenous stable isotope infusion. A second Teflon catheter will be inserted in a heated dorsal hand vein of the contra lateral arm and placed in a hot-box (60°C) for arterialized blood sampling (56). Following basal blood collection (t=-150 min), the plasma phenylalanine and tyrosine pools will be primed with a single intravenous dose of both tracers, after which continuous tracer infusion will be started that will be administered until the end of the test day (8½ h). After resting in a supine position for 90 min, a second arterialized blood sample will be drawn and a muscle biopsy will be collected from the *vastus lateralis* muscle (t=-120 min). Arterialized blood samples (8 mL) will be collected at t= -90, -60, -30, and 0 min during the postabsorptive (fasted) period with a second muscle biopsy taken from the same at t=0 min, marking the end of the postabsorptive period. Subjects will then receive the test-drink containing 20 g intrinsically labelled casein. Arterialized blood samples (8 ml) will be

collected at t= 15, 30, 45, 60, 90 and 120 min during the postprandial (fed) period. The third muscle biopsy is then taken from a new incision, 5 cm distal from the first incision, from the same leg at t=120 min. Subsequently, arterialized blood samples (8 ml) will be collected at t=150, 180, 210, 240, 270, 300 min. Finally, at 300 min a fourth muscle biopsy will be taken from the same incision as the last biopsy. In total, four muscle biopsies will be taken through two separate incisions. At the end of the test day, subjects will receive a standard lunch before they go home.

Muscle biopsies

During the test day four muscle biopsies will be obtained. Four biopsies are necessary to be able to measure mixed muscle fractional synthetic rate in a post-absorptive as well as in the post-prandial state. Biopsies will be obtained from the middle region of the vastus lateralis muscle (15 cm above the patella) and approximately 2 cm below entry through the fascia by the percutaneous needle biopsy technique described by Bergström et al. (57), using a modified needle (Maastricht Instruments). With this instrument, better results are obtained for sample size, whereas subjects' discomfort is minimized. In short, skin and muscle fascia will be locally anesthetized using 2% xylocaine. After 10 minutes a small incision will be made in skin and fascia after which the biopsy needle will be introduced in the muscle. Vacuum will be applied to the needle and, with the needle kept *in situ*, several small muscle samples (total ± 50-80 mg) will be taken. Following the muscle biopsy, the skin will be closed using a Steristrip® and covered by Tegaderm® after which a pressure bandage will be applied (Acrylastic®).

The percutaneous needle biopsy technique was introduced within the Department of Human Movement Sciences by Profs. Hans Keizer and Harm Kuipers more than 25 years ago. Since this introduction, the technique has been successfully used to perform numerous human *in vivo* studies focusing on skeletal muscle metabolism within the Department of Human Movement Sciences (for review see (58, 59)). In addition, recently approved MEC proposals continue to combine the use of continuous tracer infusion and muscle biopsy technique in young (MEC 09-3-080, MEC 09-3-054) and elderly men (MEC 07-3-086, MEC 09-3-080, MEC 09-3-054, MEC 09-3-078, MEC 10-3-065, MEC 10-3-80). Based on previous experience, discomfort for the subjects with the technique used in our laboratory is negligible.

### 6.4 Withdrawal of individual subjects

Subjects can leave the study at any time for any reason if they wish to do so without any consequences. The investigator can decide to withdraw a subject from the study for urgent medical reasons.

### 6.5 Replacement of individual subjects after withdrawal

New subjects will replace subjects that are withdrawed from the study.

### 6.6 Follow-up of subjects withdrawn from treatment

Subjects withdrawn from treatment will not be followed-up after eventually handing over responsibilities to medical personnel.

### 6.7 Premature termination of the study

In case the safety and wellbeing of the subject is in danger, or there are non-expected disadvantages of the participation for the subject, the subject will directly be excluded from the research. There are no risks connected to the premature termination of the study.

In case of premature termination or suspension of the trial for any reason, the investigator will inform:

- The regulatory authorities (MEC)
- The subject, assuring him or her appropriate treatment and follow up

# 7. SAFETY REPORTING

## 7.1 Section 10 WMO event

In accordance to section 10, subsection 1, of the WMO, the investigator will inform the subjects and the reviewing accredited METC if anything occurs, on the basis of which it appears that the disadvantages of participation may be significantly greater than was foreseen in the research proposal. The study will be suspended pending further review by the accredited METC, except insofar as suspension would jeopardise the subjects' health. The investigator will take care that all subjects are kept informed.

## 7.2 Adverse and serious adverse events

Adverse events are defined as any undesirable experience occurring to a subject during the study, whether or not considered related to the experimental treatment. All adverse events reported spontaneously by the subject or observed by the investigator or his staff will be recorded.

A serious adverse event is any untoward medical occurrence or effect that at any dose:

- results in death;
- is life threatening (at the time of the event);
- requires hospitalisation or prolongation of existing inpatients' hospitalisation;
- results in persistent or significant disability or incapacity;
- is a congenital anomaly or birth defect;
- is a new event of the trial likely to affect the safety of the subjects, such as an unexpected outcome of an adverse reaction, lack of efficacy of an IMP used for the treatment of a life threatening disease, major safety finding from a newly completed animal study, etc.

All SAEs will be reported through the web portal *ToetsingOnline* to the accredited METC that approved the protocol, within 15 days after the sponsor has first knowledge of the serious adverse reactions..

SAEs that result in death or are life threatening should be reported expedited. The expedited reporting will occur not later than 7 days after the responsible investigator has first knowledge of the adverse reaction. This is for a preliminary report with another 8 days for completion of the report.

## 7.3 Follow-up of adverse events

All adverse events will be followed until they have abated, or until a stable situation has been reached. Depending on the event, follow up may require additional tests or medical procedures as indicated, and/or referral to the general physician or a medical specialist.

# 8. STATISTICAL ANALYSIS

## 8.1 Descriptive statistics

All the data will be collected and kept in Microsoft Excel. Means and standard error of the mean (36) will be calculated and plotted in either bar of line graphs (when appropriate).

## 8.2 Univariate analysis

The Statistical Package for the Social Sciences (SPSS) will be used to perform univariate analyses. Differences in energy expenditure, substrate oxidation, (muscle) protein synthesis and sleep between the two conditions (and baseline measurement) will be tested with one-way ANOVA with Bonferroni post hoc corrections. Changes in VAS ratings over time between and within groups will be tested with repeated-measures ANOVA with Bonferroni post hoc corrections. Multiple linear regression analyses will be performed to determine between subjects effects. All statistical tests will be two-sided and differences will be considered statistical significant if $p<.05$.

## 8.3 Multivariate analysis

Multiple linear regression analyses will be performed to determine the independent contribution of gender, age and BMI on energy expenditure, substrate oxidation, (muscle) protein synthesis and sleep. All statistical tests will be two-sided and differences will be considered statistical significant if $p<.05$.

# 9. ETHICAL CONSIDERATIONS

## 9.1 Regulation statement

The study will be conducted according to the principles of the Declaration of Helsinki (9th version, October 2008, Seoul) and in accordance with the Dutch version of the Medical Research Involving Human Subjects Act (in Dutch: Wet Medisch-wetenschappelijk Onderzoek met mensen; WMO). Before the study will start, the Medical Ethics Committee of Maastricht University will approve the protocol and all relevant documents.

## 9.2 Recruitment and consent

Subjects will be recruited by advertisements in local newspapers and on notice boards at the Maastricht University. They will be informed about the study by reading the provided written 'Subject information'. The researchers will orally check their comprehensiveness. An independent person who is well informed about the study could eventually provide additional information, and subjects will be referred to the CCMO brochure 'General information for research participants' that contains general information about medical-scientific research. After being well-informed subjects will get one week before they have to decide to participate in the study. All subjects will confirm their approval for participation by signing an informed consent form at the start of the screening.

## 9.3 Benefits and risks assessment, group relatedness

Anthropometric measurements, performed during the screening, will not be invasive for the subjects. Blood sampling via venapunction is limited to one sample during the screening and one sample per day in the respiration chamber. There are no side effects, except from a minor risk of bruising. Insertion of the catheters in a vein is comparable to venapunction and here as well the only risk is a small local hematoma. This is the same for the muscle biopsy. The incision made for obtaining the muscle biopsy will be done by an experienced physician and will heal completely. The research group of Prof. Dr. L. van Loon has extensive experience with taking muscle biopsies. During the follow up several days after taking the biopsy no complications have been reported.

The test beverages contain tested normal nutritional ingredients and for this reason do not form any health risks. The labeled amino acids tracers applied in this experiment are not radioactive and are completely safe. The production of the tracers for intravenous administration will occur in a sterile environment according to GMP guidelines.

Deuterium is an isotope of water that naturally appears in the body. Drinking it does not expose the subject to any risks. Urine sampling will be done in urine bottles added with diluted HCl, which might pose a risk for the subjects. However, subjects will be carefully

instructed on how to handle the bottles to reduce these risks. MRI and MRS are safe, non-invasive procedures with no ionizing radiation and these procedures do not expose the subjects to any risks if the exclusion criteria (no electronic implants, no pacemakers, no metal fragments in the eyes, skin or body) are well respected. Additionally, there are no risks for the subject in consuming any of the provided meals, because people with certain food allergies are excluded for participation and all food items will be commercially available in normal Dutch supermarkets. Studies in the respiratory chamber will be conducted using standard operating procedures. A pair of subjects will always participate in the study at the same time and therefore they will never be alone. The subjects will be able to contact the investigators during the entire night. In addition, they will be able to get out of the chamber at any time they feel uncomfortable. A 30En% protein or a 5En% protein diet for 12 weeks will not have any side effects on the subject's health.
This study does not have any benefits for the subjects themselves, but will give possible new knowledge for treatment of obesity.

### 9.4 Compensation for injury

The sponsor/investigator has a liability insurance which is in accordance with article 7, subsection 6 of the WMO.

The sponsor (also) has an insurance which is in accordance with the legal requirements in the Netherlands (Article 7 WMO and the Measure regarding Compulsory Insurance for Clinical Research in Humans of 23th June 2003). This insurance provides cover for damage to research subjects through injury or death caused by the study.

1. € 450.000,-- (i.e. four hundred and fifty thousand Euro) for death or injury for each subject who participates in the Research;
2. € 3.500.000,-- (i.e. three million five hundred thousand Euro) for death or injury for all subjects who participate in the Research;
3. € 5.000.000,-- (i.e. five million Euro) for the total damage incurred by the organisation for all damage disclosed by scientific research for the Sponsor as 'verrichter' in the meaning of said Act in each year of insurance coverage.

The insurance applies to the damage that becomes apparent during the study or within 4 years after the end of the study.

### 9.5 Incentives

Subjects will receive a financial compensation of 75 euro per 24 hours. They will receive 450 euro in total after completion of the study. For the additional test day subjects receive 100 euro extra, this means a total of 550 euro._Possible travel expenses (maximum € 0.19 per km) will be reimbursed.

# 10. ADMINISTRATIVE ASPECTS AND PUBLICATION

## 10.1 Handling and storage of data and documents

Subjects' personal information will be confidentially handled during the study, according to the Dutch Personal Data Protection Act (Wet bescherming persoonsgegevens; Wbp). Results will be linked to the intervention and not to individual subjects. Subject numbers (e.g. number 1) in combination with treatment codes (5En% protein, code A; 30En% protein, code B) will be used throughout the study. Only the researchers are familiar with subject information, so privacy of personal information is guaranteed. If desirable, subjects will be informed about their personal study results at the end of the study. Collected body material (urine and blood) will be destroyed after publication of the results. Personal data will be kept for a period of 15 year, which is the legal time to keep medical data. Hereafter the data will be destroyed.

## 10.2 Amendments

Amendments are changes made to the research after a favourable opinion by the accredited METC has been given. All amendments will be notified to the METC that gave a favourable opinion.

A 'substantial amendment' is defined as an amendment to the terms of the METC application, or to the protocol or any other supporting documentation, that is likely to affect to a significant degree:

- the safety or physical or mental integrity of the subjects of the trial;
- the scientific value of the trial;
- the conduct or management of the trial; or
- the quality or safety of any intervention used in the trial.

All substantial amendments will be notified to the METC and to the competent authority.

Non-substantial amendments will not be notified to the accredited METC and the competent authority, but will be recorded and filed by the sponsor.

## 10.3 Annual progress report

The sponsor/investigator will submit a summary of the progress of the trial to the accredited METC once a year. Information will be provided on the date of inclusion of the first subject, numbers of subjects included and numbers of subjects that have completed the trial, serious adverse events/ serious adverse reactions, other problems, and amendments.

### 10.4 End of study report

The investigator will notify the accredited METC and the competent authority of the end of the study within a period of 90 days. The end of the study is defined as the last patient's last visit.

In case the study is ended prematurely, the sponsor will notify the accredited METC and the competent authority within 15 days, including the reasons for the premature termination. Within one year after the end of the study, the investigator/sponsor will submit a final study report with the results of the study, including any publications/abstracts of the study, to the accredited METC and the Competent Authority.

### 10.5 Public disclosure and publication policy

The CCMO Statement publication policy will be followed to publish research results after the end of the study.

## 11. REFERENCES


1. Minet-Ringuet J, Le Ruyet PM, Tome D, Even PC. A tryptophan-rich protein diet efficiently restores sleep after food deprivation in the rat. Behav Brain Res 2004;152:335-40.
2. Haslam DW, James WP. Obesity. Lancet 2005;366:1197-209.
3. Aucott LS. Influences of weight loss on long-term diabetes outcomes. Proc Nutr Soc 2008;67:54-9.
4. Repas TB. Challenges and strategies in managing cardiometabolic risk. J Am Osteopath Assoc 2007;107:S4-11.
5. Stubbs RJ, Mazlan N, Whybrow S. Carbohydrates, appetite and feeding behavior in humans. J Nutr 2001;131:2775S-2781S.
6. Bray GA, Popkin BM. Dietary fat intake does affect obesity! Am J Clin Nutr 1998;68:1157-73.
7. Simpson SJ, Batley R, Raubenheimer D. Geometric analysis of macronutrient intake in humans: the power of protein? Appetite 2003;41:123-40.
8. Tappy L. Thermic effect of food and sympathetic nervous system activity in humans. Reprod Nutr Dev 1996;36:391-7.
9. Poppitt SD, McCormack D, Buffenstein R. Short-term effects of macronutrient preloads on appetite and energy intake in lean women. Physiol Behav 1998;64:279-85.
10. Eisenstein J, Roberts SB, Dallal G, Saltzman E. High-protein weight-loss diets: are they safe and do they work? A review of the experimental and epidemiologic data. Nutr Rev 2002;60:189-200.
11. Lejeune MP, Westerterp KR, Adam TC, Luscombe-Marsh ND, Westerterp-Plantenga MS. Ghrelin and glucagon-like peptide 1 concentrations, 24-h satiety, and energy and substrate metabolism during a high-protein diet and measured in a respiration chamber. Am J Clin Nutr 2006;83:89-94.
12. Westerterp-Plantenga MS, Rolland V, Wilson SA, Westerterp KR. Satiety related to 24 h diet-induced thermogenesis during high protein/carbohydrate vs high fat diets measured in a respiration chamber. Eur J Clin Nutr 1999;53:495-502.
13. Van Cauter E, Spiegel K, Tasali E, Leproult R. Metabolic consequences of sleep and sleep loss. Sleep Med 2008;9 Suppl 1:S23-8.
14. Knutson KL, Spiegel K, Penev P, Van Cauter E. The metabolic consequences of sleep deprivation. Sleep Med Rev 2007;11:163-78.
15. Knutson KL. Impact of sleep and sleep loss on glucose homeostasis and appetite regulation. Sleep Med Clin 2007;2:187-197.
16. Nedeltcheva AV, Kilkus JM, Imperial J, Schoeller DA, Penev PD. Insufficient sleep undermines dietary efforts to reduce adiposity. Ann Intern Med;153:435-41.
17. Lindseth G, Lindseth P, Thompson M. Nutritional Effects on Sleep. West J Nurs Res.
18. Cubero J, Valero V, Sanchez J, et al. The circadian rhythm of tryptophan in breast milk affects the rhythms of 6-sulfatoxymelatonin and sleep in newborn. Neuro Endocrinol Lett 2005;26:657-61.
19. Lejeune MP, Kovacs EM, Westerterp-Plantenga MS. Additional protein intake limits weight regain after weight loss in humans. Br J Nutr 2005;93:281-9.
20. Westerterp-Plantenga MS, Lejeune MP, Nijs I, van Ooijen M, Kovacs EM. High protein intake sustains weight maintenance after body weight loss in humans. Int J Obes Relat Metab Disord 2004;28:57-64.
21. Skov AR, Toubro S, Ronn B, Holm L, Astrup A. Randomized trial on protein vs carbohydrate in ad libitum fat reduced diet for the treatment of obesity. Int J Obes Relat Metab Disord 1999;23:528-36.
22. Arsenault BJ, Beaumont EP, Despres JP, Larose E. Mapping body fat distribution: A key step towards the identification of the vulnerable patient? Ann Med.
23. Schrauwen-Hinderling VB, Hesselink MK, Schrauwen P, Kooi ME. Intramyocellular lipid content in human skeletal muscle. Obesity (Silver Spring) 2006;14:357-67.

24. Koopman R, Walrand S, Beelen M, et al. Dietary protein digestion and absorption rates and the subsequent postprandial muscle protein synthetic response do not differ between young and elderly men. J Nutr 2009;139:1707-13.
25. Pennings B, Boirie Y, Senden JM, Gijsen AP, Saris WH, van Loon LJ. Whey protein stimulates postprandial muscle protein accretion more effectively than casein and casein hydrolysate in elderly men. Am J Clin Nutr 2011;in press.
26. Pennings B, Koopman R, Beelen M, Senden JM, Saris WH, van Loon LJ. Exercising before protein intake allows for greater use of dietary protein-derived amino acids for de novo muscle protein synthesis in both young and elderly men. Am J Clin Nutr 2011.
27. van Loon LJ, Boirie Y, Gijsen AP, et al. The production of intrinsically labeled milk protein provides a functional tool for human nutrition research. J Dairy Sci 2009;92:4812-22.
28. Stunkard AJ, Messick S. The three-factor eating questionnaire to measure dietary restraint, disinhibition and hunger. J Psychosom Res 1985;29:71-83.
29. Baecke JA, Burema J, Frijters JE. A short questionnaire for the measurement of habitual physical activity in epidemiological studies. Am J Clin Nutr 1982;36:936-42.
30. Markus CR, Olivier B, de Haan EH. Whey protein rich in alpha-lactalbumin increases the ratio of plasma tryptophan to the sum of the other large neutral amino acids and improves cognitive performance in stress-vulnerable subjects. Am J Clin Nutr 2002;75:1051-6.
31. Wolfe RR. Radioactive and stable isotope tracers in biomedicine: principles and practice of kinetic analysis. New York: Wiley-Liss 1992.
32. Koopman R, Beelen M, Stellingwerff T, et al. Coingestion of carbohydrate with protein does not further augment postexercise muscle protein synthesis. Am J Physiol Endocrinol Metab 2007;293:E833-42.
33. Koopman R, Crombach N, Gijsen AP, et al. Ingestion of a protein hydrolysate is accompanied by an accelerated in vivo digestion and absorption rate when compared with its intact protein. Am J Clin Nutr 2009;90:106-15.
34. Koopman R, Pannemans DL, Jeukendrup AE, et al. Combined ingestion of protein and carbohydrate improves protein balance during ultra-endurance exercise. Am J Physiol Endocrinol Metab 2004;287:E712-20.
35. Koopman R, Verdijk L, Manders RJ, et al. Co-ingestion of protein and leucine stimulates muscle protein synthesis rates to the same extent in young and elderly lean men. Am J Clin Nutr 2006;84:623-32.
36. Koopman R, Verdijk LB, Beelen M, et al. Co-ingestion of leucine with protein does not further augment post-exercise muscle protein synthesis rates in elderly men. Br J Nutr 2008;99:571-80.
37. Koopman R, Wagenmakers AJ, Manders RJ, et al. Combined ingestion of protein and free leucine with carbohydrate increases postexercise muscle protein synthesis in vivo in male subjects. Am J Physiol Endocrinol Metab 2005;288:E645-53.
38. Manders RJ, Koopman R, Beelen M, et al. The muscle protein synthetic response to carbohydrate and protein ingestion is not impaired in men with longstanding type 2 diabetes. J Nutr 2008;138:1079-85.
39. Brouwer E. On simple formulae for calculating the heat expenditure and the quantities of carbohydrate and fat oxidized in metabolism of men and animals, from gaseous exchange (Oxygen intake and carbonic acid output) and urine-N. Acta Physiol Pharmacol Neerl 1957;6:795-802.
40. Siri WE. Body composition from fluid spaces and density: analysis of methods. 1961. Nutrition 1993;9:480-91; discussion 480, 492.
41. van Marken Lichtenbelt WD, Westerterp KR, Wouters L. Deuterium dilution as a method for determining total body water: effect of test protocol and sampling time. Br J Nutr 1994;72:491-7.
42. Albrecht RR, Ewing SJ. Standardizing the administration of the Profile of Mood States (POMS): development of alternative word lists. J Pers Assess 1989;53:31-9.
43. Tenenbaum G, Furst D, Weingarten G. A statistical reevaluation of the STAI anxiety questionnaire. J Clin Psychol 1985;41:239-44.

44. Stevens LA, Coresh J, Feldman HI, et al. Evaluation of the modification of diet in renal disease study equation in a large diverse population. J Am Soc Nephrol 2007;18:2749-57.
45. Levey AS, Bosch JP, Lewis JB, Greene T, Rogers N, Roth D. A more accurate method to estimate glomerular filtration rate from serum creatinine: a new prediction equation. Modification of Diet in Renal Disease Study Group. Ann Intern Med 1999;130:461-70.
46. Schoffelen PF, Westerterp KR, Saris WH, Ten Hoor F. A dual-respiration chamber system with automated calibration. J Appl Physiol 1997;83:2064-72.
47. Westerterp KR, Wilson SA, Rolland V. Diet induced thermogenesis measured over 24h in a respiration chamber: effect of diet composition. Int J Obes Relat Metab Disord 1999;23:287-92.
48. Tintera J, Harantova P, Suchanek P, et al. Quantification of intra-abdominal fat during controlled weight reduction: assessment using the water-suppressed breath-hold MRI technique. Physiol Res 2004;53:229-34.
49. Schrauwen-Hinderling VB, Schrauwen P, Hesselink MK, et al. The increase in intramyocellular lipid content is a very early response to training. J Clin Endocrinol Metab 2003;88:1610-6.
50. Stefan N, Machann J, Schick F, et al. New imaging techniques of fat, muscle and liver within the context of determining insulin sensitivity. Horm Res 2005;64 Suppl 3:38-44.
51. Wolfe RR. Radioactive and Stable Isotope Tracers in Biomedicine: Principles and Practice of Kinetic Analysis New York: Wiley-Liss, 1992.
52. Levine J, Melanson EL, Westerterp KR, Hill JO. Tracmor system for measuring walking energy expenditure. Eur J Clin Nutr 2003;57:1176-80.
53. Westerterp KR, Speakman JR. Physical activity energy expenditure has not declined since the 1980s and matches energy expenditures of wild mammals. Int J Obes (Lond) 2008;32:1256-63.
54. Harris JA, Benedict FG. A Biometric Study of Human Basal Metabolism. Proc Natl Acad Sci U S A 1918;4:370-3.
55. Westerterp KR, Kester AD. Physical activity in confined conditions as an indicator of free-living physical activity. Obes Res 2003;11:865-8.
56. Abumrad NN, Rabin D, Diamond MP, Lacy WW. Use of a heated superficial hand vein as an alternative site for the measurement of amino acid concentrations and for the study of glucose and alanine kinetics in man. Metabolism 1981;30:936-940.
57. Bergstrom J. Percutaneous needle biopsy of skeletal muscle in physiological and clinical research. Scand J Clin Lab Invest 1975;35:609-16.
58. Koopman R, van Loon LJC. Aging, exercise and muscle protein metabolism. J Appl Physiol 2009.
59. Koopman R, Saris WH, Wagenmakers AJ, van Loon LJ. Nutritional interventions to promote post-exercise muscle protein synthesis. Sports Med 2007;37:895-906.
